# Phylomorphometrics reveal ecomorphological convergence in pea crab carapace shapes (Brachyura, Pinnotheridae)

**DOI:** 10.1002/ece3.9744

**Published:** 2023-01-16

**Authors:** Werner de Gier

**Affiliations:** ^1^ Naturalis Biodiversity Center Leiden The Netherlands; ^2^ Groningen Institute for Evolutionary Life Sciences University of Groningen Groningen The Netherlands

**Keywords:** ecomorphology, morphometry, parasitism, phylomorphospace, symbiosis

## Abstract

Most members of the speciose pea crab family (Decapoda: Brachyura: Pinnotheridae) are characterized by their symbioses with marine invertebrates in various host phyla. The ecology of pea crabs is, however, understudied, and the degree of host dependency of most species is still unclear. With the exception of one lineage of ectosymbiotic echinoid‐associated crabs, species within the subfamily Pinnotherinae are endosymbionts, living within the body cavities of mollusks, ascidians, echinoderms, and brachiopods. By contrast, most members of the two other subfamilies are considered to have an ectosymbiotic lifestyle, sharing burrows and tubes with various types of worms and burrowing crustaceans (inquilism). The body shapes within the family are extremely variable, mainly in the width and length of the carapace. The variation of carapace shapes in the family, focusing on pinnotherines, is mapped using landmark‐based morphometrics. Mean carapace shapes of species groups (based on their host preference) are statistically compared. In addition, a phylomorphometric approach is used to study three different convergence events (across subfamilies; between three genera; and within one genus), and link these events with the associated hosts.

## Graphical abstract

The speciose pea crab family is studied using landmark‐based morphometrics. Mean carapace shapes of species groups, based on their host associations, are compared and visualized. Additionally, with the inclusion of a phylogeny, three morphological convergence events are studied and statistically tested in a phylomorphometric framework.

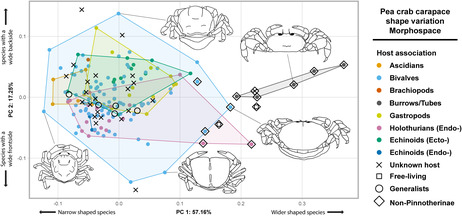

## 1 INTRODUCTION

Most pea crab species (Crustacea: Decapoda: Brachyura: Pinnotheridae) are characterized by their symbiotic lifestyle, often thought to be obligate parasites or commensals (Baeza, 2015). The degree of host dependency is, however, still unclear for most species. The species within the subfamily Pinnotherinae are known to be endo‐ or ectosymbiotic inhabitants of bivalves, polyplacophorans, gastropods, holothurians, echinoids, brachiopods, and ascidians (de Gier & Becker, 2020). Although some species have a broad host spectrum spanning across various phyla, most species of Pinnotherinae are considered to be specialists. This means that these species are only inhabiting specific genera or orders within a host phylum. Several species of the two smaller subfamilies (Pinnixinae and Pinnixulalinae) are also found in bivalve, or in/on holothurian hosts in their adult stages, but most are considered to be free‐living or tube/burrow‐dwelling, sharing this habitat with a worm, ghost shrimp, or mud lobster as a host (inquilism; see Palacios Theil et al., 2016; Palacios Theil & Felder, 2020a; Schmitt et al., 1973; Zmarzly, 1992). It is worth noting that also in these subfamilies, the host specificity is understudied and some species now considered to be free‐living might have unknown host associations (McDermott, 2009).

Pinnotherids have evolved a wide range of ecomorphological adaptations that could be linked to their presumed host choice (described for the subfamily Pinnotherinae in de Gier & Becker, 2020). These include: (A) several types of setae on the walking legs and claws used for feeding, swimming, or camouflaging; (B) asymmetry and widening of the walking legs' segments for feeding purposes and/or grip within the host (or host tube/burrow); and (C) various ornamentations, setaetion and colouration patterns, shape differences, and a variation of thickness of the carapaces in order to fit inside their hosts or to blend with their hosts' colouration (de Gier & Becker, 2020, and references therein for examples of adapted species). In addition, in sexual dimorphic species, females have evolved an enlarged pleon to carry eggs, making them almost immobile and very vulnerable to predation if they ever leave their host (Baeza, 2015). This is likely a consequence of living hidden within a host (de Gier & Becker, 2020). Although the variation in the above‐mentioned characters is most diverse in the speciose pea crab family, similar adaptations to endo‐ or ectosymbiotic lifestyles can be found in other brachyuran (“true” crab) taxa (Castro, 2015; Serène, 1961). For example, coral‐inhabiting parasitic gall crabs (Cryptochiridae) share a strong sexual dimorphism and enlarged pleons in females (Vehof et al., 2016). Adaptive walking‐leg morphology and host‐specific camouflage can be found in the echinoderm‐associated Eumedoninae subfamily (Pilumnidae) and the coral‐associated Domeciidae, Tetraliidae, and Trapeziidae (Castro et al., 2004; Chia & Ng, 2000; Ng & Jeng, 1999; Števčić et al., 1988). In addition, symbiotic members of the families Portunidae (e.g., the cnidarian‐ and echinoderm‐associated members of *Lissocarcinus* Adams & White, 1849 and *Caphyra* Guérin, 1832) and Varunidae (the burrow‐dwelling *Sestrostoma* Davie & Ng, 2007) have also evolved adapted walking‐leg dactyli, carapace shapes, and cryptic camouflage (Davie & Ng, 2007; Evans, 2018). Two clades of endosymbiotic shrimp (Caridea: Palaemonidae), which share a similar lifestyle with ascidian‐ and bivalve‐associated pea crabs, evolved similar morphological features, mainly in the round, swollen shape of their abdomen and smooth carapace (de Gier et al., 2022; Fransen, 1994).

Most of the ecomorphological adaptations mentioned above have only briefly been described in taxonomic and phylogenetic literature (Campos, 1996a, 2016; Manning, 1993; Palacios Theil & Felder, 2020b), and were not directly studied with respect to the crabs' host choice. In a large‐scale study, Laughlin (1981) mentioned that various pinnixine pea crabs (mainly including species then attributed to *Pinnixa* White, 1846) have a much wider carapace than the studied pinnotherines (in this case, species of *Pinnotheres* Bosc, 1801), and linked this character to their biology. More recently, Hultgren et al. (2022) analyzed the relationship between the host choice of a wide range of pea crabs and their carapace size ratios, considering also their phylogenetic positions. In this way, convergence in carapace shapes could be studied. They did this by testing the aspect ratios of 149 species, 59 of which had known phylogenetic positions (see Palacios Theil et al., 2016).

The present study elaborates on the analyses by Hultgren et al. (2022), by using additional morphometrics to investigate the relationship between the adult female carapace shape, and the host choice, focusing on all currently included pinnotherine members. A phylomorphospace approach will be used, including both symbiotic, as well as free‐living outgroup species from the two other subfamilies. This projection of the phylogeny should reveal clusters and convergence patterns in the data (Stayton, 2015), indicating that the colonization of similar host phyla has led to analogous carapace shapes in the evolution of pea crabs.

## 2 MATERIAL AND METHODS

### 2.1 Selection of illustrations

**FIGURE 1:**
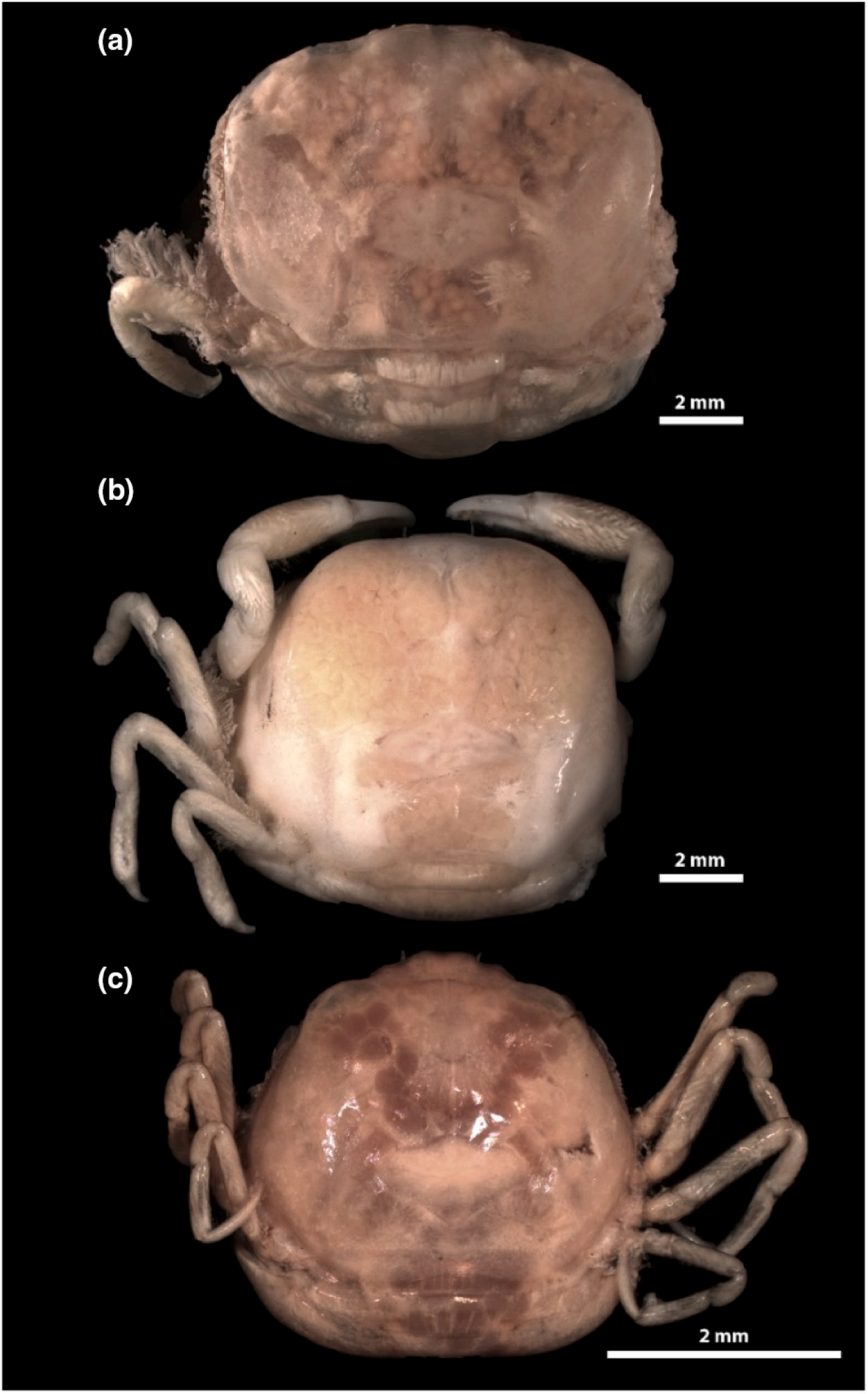
Three newly made photographs of pinnotherine species, which were used in the current analyses. (a) *Mesotheres serrei* (Rathbun, 1909) (SMF 9435) (all walking legs but one, and both claws were severed). (b) *Pinnotheres pectunculi* Hesse, 1872 (SMF 34002) (right side walking legs were severed). (c) *Arcotheres quadratus* (Rathbun, 1909) (ZMA.CRUS.D.242266).

Similarly to the methods described by Hultgren et al. (2022), published illustrations of 181 pea crab species (in particular Pinnotherinae; see below) were collected through an extensive literature search. Available dorsal views of adult female carapaces (independently of the number of pereiopods depicted in the illustration) were selected for the analyses due to their often obligatory symbiosis, being restricted to remain inside their host. By contrast, males often leave their host, and juveniles have been found to switch hosts multiple times before reaching their adult stages (de Gier & Becker, 2020). In addition, one rarely figured species, and two species without previously published illustrations were photographed using a Leica M165c stereo microscope with a Leica DFC420 microscope‐mounted camera, using Leica LAS 4.4 software and Helicon Focus stacking software. These species are *Mesotheres serrei* (Rathbun, 1909) (SMF 9435) and *Pinnotheres pectunculi* Hesse, 1872 (SMF 34002) from the Senckenberg Forschungsinstitut und Naturmuseum collection (Frankfurt, Germany), and *Arcotheres quadratus* (Rathbun, 1909) (ZMA.CRUS.D.242266) from the collection of Naturalis Biodiversity Center (Figure 1).

From all the currently recognized species of Pinnotherinae (excluding two genera discussed below), 168 species could be included in the study. Thirty‐five species were excluded due to the absence of illustrations of the dorsal view of adult female crabs (Appendix S1). To cover the morphological variation of the non‐pinnotherine pea crabs, outgroup illustrations were selected of ten species from the other pea crab subfamilies. Nine were selected from Pinnixinae and one from Pinnixulalinae. A representative of both the genera *Sakaina* Serène, 1964 and *Parapinnixa* Holmes, 1895 were also added but considered as outgroups, although they are still within the subfamily Pinnotherinae (WoRMS Editorial Board, 2022), as the phylogeny of Palacios Theil et al. (2016) suggests that this placement is rather questionable. The subfamily classification of pea crabs seems to be unstable and in need of further research, and therefore the subfamily status for these species will be annotated as “Pinnotherinae?”. In addition, one species with a tentative placement basal to the three subfamilies is included as an outgroup: *Tetrias fischerii* (A. Milne‐Edwards, 1867). This species has no subfamily status (WoRMS Editorial Board, 2022). Five of the outgroup species were chosen based on their recorded, or presumed endosymbiotic lifestyle: *Tetrias fischerii* is thought to be associated with bivalves (Milne‐Edwards, 1873), the pinnixines *Pinnixa barnharti* Rathbun, 1918 and *Pinnixa tumida* Stimpson, 1858 are thought to be internal symbionts of holothurians (Dai & Yang, 1991; Zmarzly, 1992), and adults of the pinnixines *Scleroplax faba* (Dana, 1851) and *Scleroplax littoralis* (Holmes, 1895) are commonly found in bivalve hosts (Zmarzly, 1992). The latter two are suggested to be morphotypes of the same species (Palacios Theil & Felder, 2020a; Zmarzly, 1992) but are treated as separate species in the analyses. *Scleroplax faba* was reported in various other host types (gastropods, ascidians, and holothurians) in juvenile specimens (Zmarzly, 1992).

Sources of illustrations used are presented as supplementary data (Appendix S2). Taxonomy follows WoRMS (WoRMS Editorial Board, 2022), taking the taxonomic decisions made after de Gier and Becker (2020) into account, mainly within *Arcotheres* Manning, 1993 (e.g., Ng & Ahyong, 2022).

### 2.2 Landmark selection and morphometrics

**FIGURE 2:**
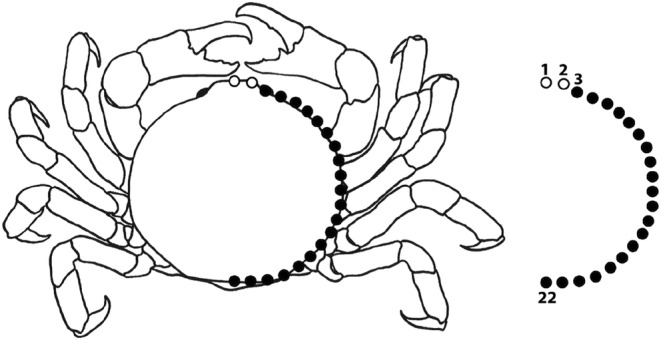
*Ostracotheres tridacnae* (Rüppell, 1830) after Ahyong (2018), with the 22 landmarks plotted on the crab's carapace. Darkened circles are part of the sliding semi‐landmark curve, whereas landmarks 3 and 22 are stationary. Setae in illustration omitted.

Collector bias is a common problem in morphometric studies when selecting landmark data (e.g., Percival et al., 2019), as is the use of nonhomologous and inconsistent datapoints (e.g., nonuniform orientations of specimens; Collins & Gazley, 2017). These problems could be evaded due to the uniformity in the orientations of illustrations used in taxonomic pea crab publications: only uniform (dorsal) orientations with visible ocular carapace ridges (cavities for the eyes) were used (with the exception of anteriorly ornamented species). Landmark (LM) selection was done to digitize the right half of the pea crabs' carapace shape, with the inclusion of three landmarks (LM 1, 3, 22), one semi‐landmark (LM 2), and 18 sliding semi‐landmarks (curve) (LM 4 to 21) along the lateral and caudal margin of the carapace (see Figure 2). Because of the lack of homologous anatomical features on the lateral curvature of pea crab carapaces, sliders were used to capture the shape variation.

1. Central tip of the rostrum

2. Halfway between LM 1 and 3

3. Central point of the ocular cavity (if the eye is not visible, the part of the carapace directly above the eye is marked)—beginning of curve 4 to 21. Curve of 18 sliding semi‐landmarks with fixed distance along the anterior, lateral, and caudal margin of the carapace 22. Most caudal tip of the carapace—end of curve

Landmark data were gathered in tpsDig2 (v. 2.31) (Rohlf, 2017) and analyzed using R v. 4.2.1 and Rstudio v. 2022.07.0 (R Core Team, 2022; RStudio Team, 2022), using the packages *geomorph* v. 4.0.4 and *ggplot2* v. 3.3.6 (Adams et al., 2022; Baken et al., 2021; Wickham, 2016). A generalized Procrustes analysis was performed to scale, transform and rotate all images, or a subset of the images for morphospace analyses. In this way, the scale was set to “uniform”, also to take into account potential uniform swelling due to preservation in ethanol. A Procrustes pairwise (M)ANOVA with a residual randomization permutation procedure (1000 permutations, RRPP; Collyer & Adams, 2021) was performed to find significant differences in the mean shape data based on the host associations of the specimens. The Procrustes pairwise ANOVA test compares the mean shapes of two specified groups by checking the relative distance between these two shapes (Goodall, 1991). In this way, it explains if the differences between the groups are large enough (e.g., significant) in comparison to the variation within the groups. The full dataset, including outgroup species, as well as a subset only including the “true” Pinnotherinae (i.e., excluding the members of *Parapinnixa* and *Sakaina*), was tested and compared. All species were labeled considering their host association: bivalve‐, gastropod‐, holothurian‐, ascidian‐, echinoid‐, and brachiopod‐associated, and tube/burrow‐dwelling. The echinoderm associates were separated based on the external or internal nature of their symbiosis (ecto‐ or endosymbiotic). In addition, two outgroup species are labeled as “free‐living”, although they could have been dislodged from their host (see McDermott, 2009; Appendix S2). Five ingroup species with a generalist multi‐phylum host range are labeled separately (de Gier & Becker, 2020). These species are: *Afropinnotheres dofleini* (Lenz in Lenz & Strunck, 1914), *Austrotheres holothuriensis* (Baker, 1907), *Nepinnotheres pinnotheres* (L., 1758), *Opisthopus transversus* Rathbun, 1894, and *Pinnaxodes major* Ortmann, 1894 (Appendix S2). Ingroup species with an unknown host association are labeled as “unknown” in the dataset. These 21 species are often rarely caught, poorly described, or have a very questionable host association. Two of these species (*Hospitotheres powelli* Manning, 1993 and *Pinnotheres taichungae* Sakai, 2000) were previously recorded as burrow‐dwelling or free‐living. It has been argued that they may have been dislodged from their host or that their host was destroyed during collection (de Gier & Becker, 2020; McDermott, 2009). Despite this, the choice was made to include them in the dataset, in order to test whether there is a predictive value in the analysis (see Discussion).

### 2.3 Phylomorphospace analyses

In order to include the available phylogenetic information of 33 species and to link this to the morphospace, a pruned (trimmed‐down) ultrametric version of the phylogeny reconstruction of Palacios Theil et al. (2016) and Hultgren et al. (2022) was used. This phylomorphospace analysis was also performed in R, using the packages *phytools* v. 1.0‐3, *ape* v. 5.6‐2, and *geiger* v. 2.0.10 (Paradis & Schliep, 2019; Pennell et al., 2014; Revell, 2012). Three convergence events were highlighted in the phylomorphospace using *ggplot2*. The phylogeny reconstruction is treated as an overlay on the presented morphospace. The branches were also used to statistically test three potential convergence events in the phylomorphospace plot. Similarity‐based measures (C_1_ to C_4_, and corresponding *p*‐values) were calculated using the R package *convevol* v. 1.3 (Stayton, 2018) as described by Stayton (2015). Examples of their uses are presented by Serb et al. (2017), Zelditch et al. (2017), Stange et al. (2018), and Grossnickle et al. (2020). A custom R‐script (Zelditch et al., 2017; Zelditch, pers. comm.) was used to run 1000 replicates to check the results from the *convevol* package. For the calculations of the C‐values, PC‐values were used from PC1 to PC3 (84.5% of the explained data).

A phylogenetically informed ANOVA (Phylogenetic Generalized Least Squares; PGLS) was performed to investigate the impact of host choice on the shape variation in the data while controlling for the independence of the residuals from the phylogeny (Adams & Collyer, 2018; Mundry, 2014). This was done for 33 species, using the procD.pgls() command in *geomorph*, with Pagel's lambda (λ) (Pagel, 1999) set at 1.0 (a high phylogenetic signal—Brownian motion model). For comparison, a regular Procrustes ANOVA/regression, without the implementation of a phylogenetic framework, was performed for the 33 species (similar to the pairwise test explained above). In both analyses, a similar RRPP approach was used as mentioned above (1000 permutations).

## 3 RESULTS

### 3.1 Morphospaces and mean shapes

**FIGURE 3:**
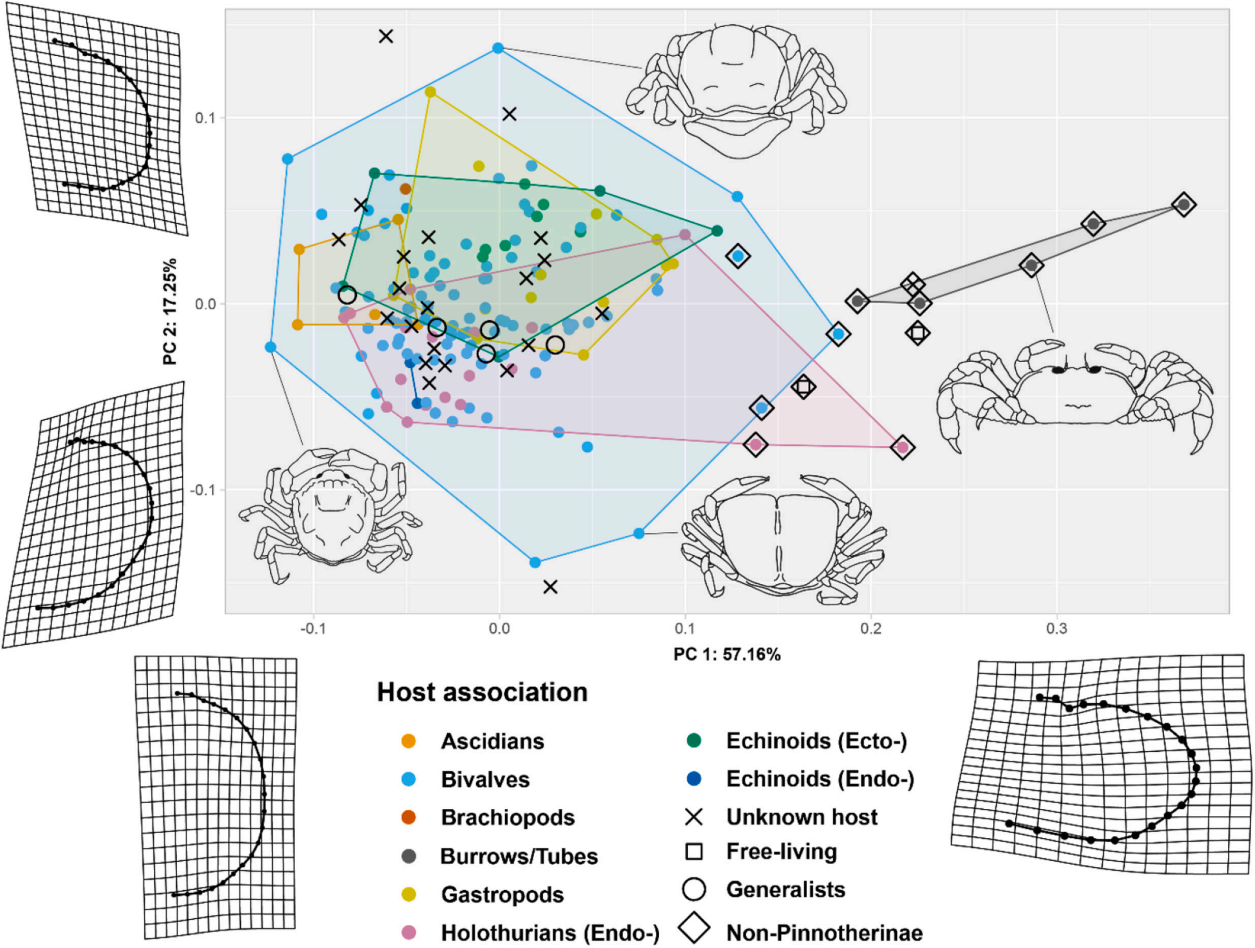
Morphospace plot showing the total variation of dorsal carapace shapes of both the in‐ and outgroups. Warps show extreme shape variation along the first and second PCs. Colors of points and convex hulls correspond to host association type, and shapes give an indication if the species have an unknown, free‐living, or generalist symbiotic lifestyle. Diamonds show the non‐pinnotherine outgroups. Illustrated species correspond to linked datapoints in morphospace: top, *Fabia tellinae* Cobb, 1973 (after Campos, 1996b); right, *Glassella floridana* (Rathbun, 1918) (after Felder & Palacios Theil, 2020a); bottom, *Durckheimia lochi* Ahyong & Brown, 2003 (after Ahyong & Brown, 2003); left, *Austrotheres pregenzeri* Ahyong, 2018 (after Ahyong, 2018) (setae in illustrations omitted; crabs not to scale).

The morphometric analyses of the scored landmarks revealed the overall variation in carapace shapes (for a morphospace plot with numbers indicating the species, see Appendix S2 (list) and S3 (figure)). When including the outgroup (non‐pinnotherinae) species, the first two of 43 principal components (PCs) explain 74.4% of the variation in the data (Figure 3). PC1 to PC11 together explain 99% of the data, meaning that the rest of the 32 PCs explain less than 1% of the data. Along these first two axes, the mean shapes change mainly in width and length: along PC1, the carapace changes from an elongated and rounder shape (PC1_min_) to a widened, angular shape, with more defined ocular cavities in dorsal view (PC1_max_). Along PC2, the widest point of the carapace seems to slightly shift from the anterolateral side (PC2_min_) to the posterolateral side (PC2_max_), meaning that the shape in the middle would have the widest point in the middle of the carapace. In addition, the rostrum seems to be much wider and more defined in specimens from the upper side of the plot (PC2_max_) (Figure 3). This also means that a perfectly round species would approximately be found in the center of this plot (0,0).

There is a clear separation between the ingroups and the outgroups, except for one outgroup species: *Tetrias fischerii*. Outgroup species with a similar host association to ingroup members (namely endosymbionts of holothurians or bivalves; see Appendix S2, S3) are closer to the ingroup than to the rest of the outgroup species, which are burrow‐ and tube‐dwelling (Figure 3). The significant difference between the point cloud of this host type and the rest of the host categories was confirmed by the pairwise Procrustes ANOVA (*p* < .01; Table 1).

**TABLE 1:** Output of pairwise Procrustes ANOVA of both datasets (with and without outgroup species included)

Host type combination	With outgroup			Without outgroup		
	*d*	UCL (95%)	Pr > *d*	*d*	UCL (95%)	Pr > *d*
Asc:Biv	0.078	0.081	0.056	0.075	0.059	0.011*
Asc:Bra	0.084	0.213	0.531	0.083	0.146	0.445
Asc:Bur/Tub	0.356	0.108	0.001*			
Asc:Ech_Ecto	0.108	0.096	0.027*	0.109	0.069	0.001*
Asc:Ech_Endo	0.080	0.147	0.397	0.081	0.110	0.219
Asc:Free	0.277	0.150	0.001*			
Asc:Gas	0.117	0.092	0.010*	0.117	0.067	0.001*
Asc:Gen	0.065	0.111	0.330	0.066	0.077	0.148
Asc:Hol_Endo	0.084	0.090	0.069	0.063	0.065	0.061
Biv:Bra	0.081	0.220	0.439	0.078	0.136	0.416
Biv:Bur/Tub	0.294	0.077	0.001*			
Biv:Ech_Ecto	0.072	0.051	0.007*	0.077	0.039	0.001*
Biv:Ech_Endo	0.063	0.121	0.422	0.061	0.096	0.310
Biv:Free	0.217	0.132	0.003*			
Biv:Gas	0.050	0.049	0.044*	0.054	0.039	0.002*
Biv:Gen	0.038	0.076	0.558	0.039	0.058	0.363
Biv:Hol_Endo	0.044	0.043	0.047*	0.044	0.034	0.005*
Bra:Bur/Tub	0.335	0.222	0.011*			
Bra:Ech_Ecto	0.086	0.214	0.446	0.084	0.137	0.361
Bra:Ech_Endo	0.114	0.235	0.369	0.112	0.159	0.261
Bra:Free	0.272	0.236	0.028*			
Bra:Gas	0.090	0.211	0.402	0.090	0.137	0.295
Bra:Gen	0.096	0.209	0.398	0.095	0.143	0.291
Bra:Hol_Ecto	0.113	0.212	0.215	0.099	0.140	0.197
Bur/Tub:Ech_Ecto	0.276	0.091	0.001*			
Bur/Tub:Ech_Endo	0.334	0.139	0.001*			
Bur/Tub:Free	0.109	0.145	0.182			
Bur/Tub:Gas	0.256	0.090	0.001*			
Bur/Tub:Gen	0.301	0.111	0.001*			
Bur/Tub:Hol_Endo	0.290	0.088	0.001*			
Ech_Ecto:Ech_Endo	0.104	0.130	0.119	0.106	0.099	0.036*
FreeL:Ech_Ecto	0.209	0.134	0.004*			
FreeL:Ech_Endo	0.249	0.175	0.007*			
FreeL:Gas	0.188	0.131	0.010*			
FreeL:Gen	0.218	0.149	0.005*			
FreeL:Hol_Endo	0.204	0.136	0.002*			
Gas:Ech_Ecto	0.054	0.066	0.141	0.058	0.052	0.021*
Gas:Ech_Endo	0.103	0.128	0.126	0.103	0.100	0.045*
Gas:Gen	0.073	0.090	0.122	0.073	0.067	0.030*
Gas:Hol_Endo	0.075	0.061	0.015*	0.084	0.048	0.001*
Gen:Ech_Ecto	0.079	0.091	0.098	0.083	0.068	0.009
Gen:Ech_Endo	0.058	0.144	0.665	0.060	0.112	0.528
Gen:Hol_Ecto	0.025	0.087	0.908	0.018	0.064	0.973
Hol_Endo:Ech_Ecto	0.086	0.061	0.006*	0.091	0.048	0.001*
Hol_Endo:Ech_Endo	0.059	0.130	0.537	0.050	0.102	0.587

*Note*: Host associations are abbreviated: Asc = Ascidians, Biv = Bivalves, Bra = Brachiopods, Bur/Tub = Burrows/Tubes, Gas = Gastropods, Hol_Endo = Holothurians (Endosymbiotic), Ech_Ecto = Echinoids (Ectosymbiotic), Ech_Endo = Echinoids (Endosymbiotic), Gen = Generalists, Free = Free‐living. *d* = distance between mean shapes, UCL (95%) = 95% upper confidence level, Pr > d = *p*‐value associated with the mean shape distances (d). Significant values (*p* < .05) are indicated by an asterisk (*).

All species of the ingroup are covered by a vast cloud of bivalve‐associated points (Figure 3; Appendix S2, S3). Although overlapping, significant results were found in the data by the pairwise ANOVA, taking all 43 PCs into account (Table 1). The five ascidian‐associated species group on the left side of the plot. Ascidian‐associated species were found to be shaped significantly different from gastropod‐associated (*p* = .010) and externally echinoid‐associated species (*p* = .027). In addition, a nearly significant difference was found between the mean shape of the ascidian‐associated species and the bivalve (*p* = .056), and internal holothurian associates (*p* = .069). Bivalve‐associated species were significantly different from gastropod associates (*p* = .044), internal associates of holothurians (*p* = .047), and external echinoid associates (*p* = .007). Between these last two groups, a significant result was found (*p* = .006). Lastly, internal holothurian associates were significantly differently shaped than gastropod associates (*p* = .015). The actual morphological differences between the carapace shapes are explained in detail below.

**FIGURE 4:**
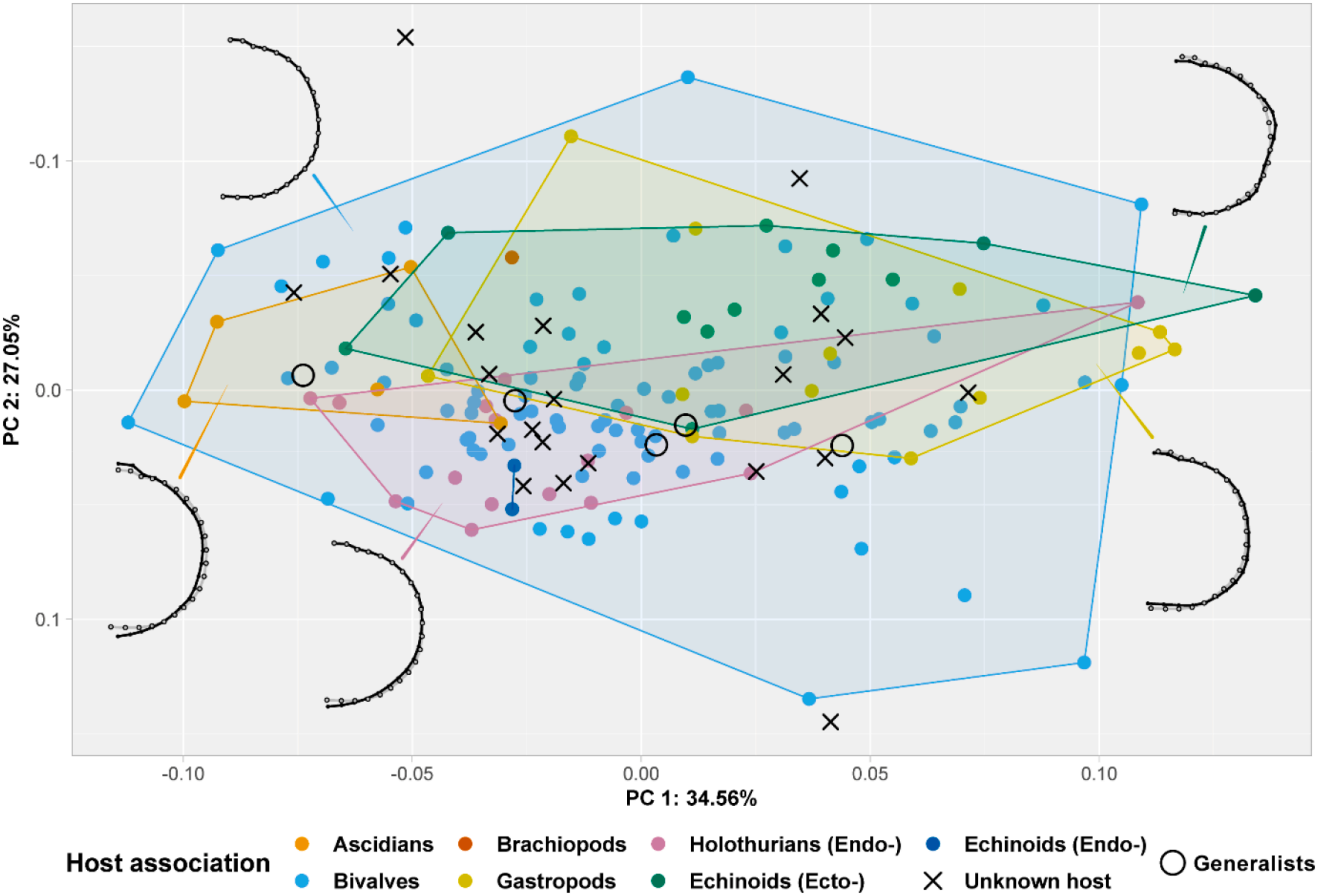
Morphospace plot showing the total variation of dorsal carapace shapes of the ingroup. Mean shapes of major host‐associated groups are plotted (black) and compared with the mean shape of the entire ingroup (gray/white). Colors of points and convex hulls correspond to host association type, and shapes give an indication if the species have an unknown or generalist symbiotic lifestyle. Note that the *y*‐axis (PC2) is flipped compared with Figure 3, and the aspect ratio is reduced to 0.5 for comparison with other plots.

When excluding the outgroup from the analyses, the bivalve‐associated convex hull overlaps all but five specimens with a known host association (Figure 4). These species (the external echinoid associate *Dissodactylus latus* Griffith, 1987, the holothurian‐associated *Holothuriophilus trapeziformis* Nauck, 1880, and the three gastropod‐associated *Mesotheres unguifalcula* (Glassel, 1936), *Orthotheres bayou* Ng & Ho, 2016, and *Orthotheres turboe* Sakai, 1969) have a broader body shape than the rest of the ingroup (i.e., a higher AR carapace aspect ratio; Hultgren et al., 2022). Running the analysis with only pinnotherine members influences the *p*‐values of the Procrustes pairwise ANOVA to be lower in all significant values from the analysis with the outgroup species included (Table 1), and the first two PCs to explain less of the calculated variation (61.6%) (in addition, the first 12 of 43 PCs explain 99% of the data). In addition, four differences between the mean shapes of the carapaces, which were in the previous analysis labeled as insignificant, were calculated and found to be significant between the following groups: ascidian and bivalve associates (*p* = .011); gastropod and external echinoid associates (*p* = .021); gastropod and internal echinoid associates (of which there are only two) (*p* = .045); and between external and internal associates of echinoids (*p* = .036). When sorted based on host association, the mean shapes of the carapaces can be calculated and projected on the mean shape of the entire dataset (Figure 4). The mean of the bivalve‐associated crab carapace shapes is almost identical to the mean shape of the entire ingroup. External echinoid‐associated species have more angular lateral sides of the carapace, while the internal symbionts are rounded. Additionally, a somewhat wider body shape can be seen in the gastropod‐associated species. The ascidian‐ and holothurian‐associated species show a more pointed posterior side of their carapace, and the widest point of the carapace is found on the anterolateral side. The bivalve‐ and gastropod‐associated species have a straight posterior side of their carapace. Lastly, the rostrum of ascidian‐associated species is slightly broader and more pronounced.

### 3.2 Phylomorphospace approach

**FIGURE 5:**
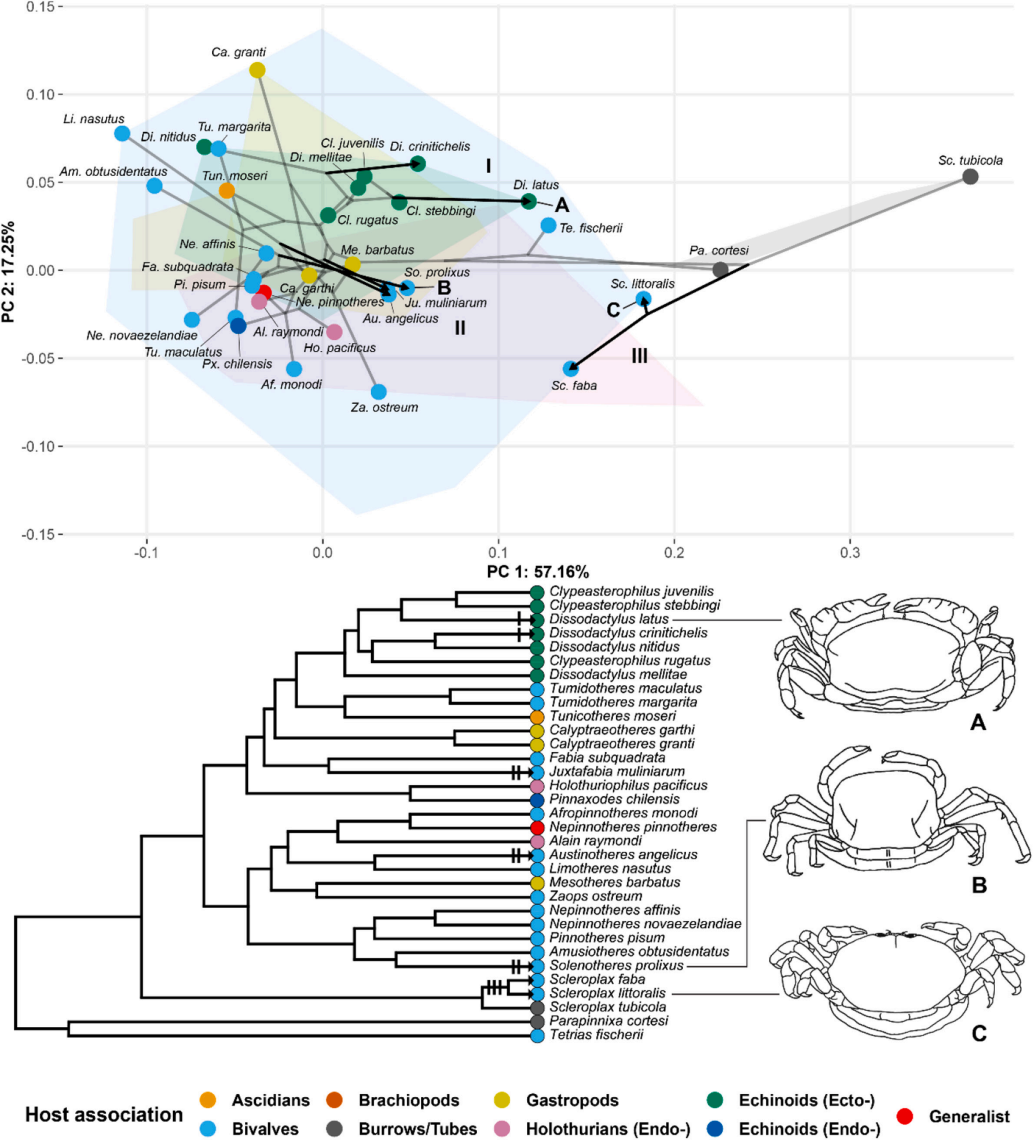
Phylomorphospace plot and the projected ultrametric phylogeny reconstruction. Specimens not included in the phylogeny are omitted in the morphospace for better readability. Colors of points and convex hulls correspond to the species' host association. PCs and corresponding shape changes along these axes same as in Figure 3. Three convergence events are highlighted with arrows in both the phylomorphospace (I to III) and the tree, of which three species (A to C) are illustrated next to the tree: A, *Dissodactylus latus* Griffith, 1987 (after Griffith, 1987); B, *Solenotheres prolixus* Ng & Ngo, 2010 (after Ng & Ngo, 2010); C, *Scleroplax littoralis* (Holmes, 1895) (after Palacios Theil & Felder, 2020a) (setae omitted in illustrations; crabs not to scale).

A pruned phylogeny tree was projected on the morphospace (Figure 3), resulting in a so‐called phylomorphospace (Figure 5). The tree “starts” in the center and to the right side of the plot with the branches containing *Tetrias fischerii* and *Parapinnixa cortesi* Thoma, Heard & Vargas, 2005, respectively. These two “ancestral” branches come together in the center of the plot, after which the tree splits into two branches, directed into opposite directions of the plot. To the right, representing Pinnixinae, the three species of *Scleroplax* Rathbun, 1894 (of which two are endosymbiotic and somewhat more round, and one is tube‐dwelling and extremely wide), and to the left the phylogeny projection stays on one side of the plot, overlapping while branching off into all the ingroup species (Pinnotherinae).

Convergence in morphology can be observed when branches split off in separate instances, but point in the same direction. Instances of presumed convergence can be observed in the plot above (Figure 5). Three examples of presumed convergence are highlighted here: within a genus (I), between three different genera (II), and between subfamilies (III). Morphological convergence within a genus is suggested here within *Dissodactylus* Smith, 1870, where *Dissodactylus latus* (Figure 5: crab A) and *Dissodactylus crinitichelis* Moreira, 1901 share a wider carapace shape than the rest of the genus. A similar shift in morphology can be found in the branches including *Solenotheres prolixus* Ng & Ngo, 2010 (Figure 5: crab B), *Juxtafabia muliniarum* (Rathbun, 1918), and *Austinotheres angelicus* (Lockington, 1877), where the evolution of three different clades resulted in almost the same wide carapace morphology. Interestingly, the two sister species of *S. prolixus* and *A. angelicus* appear to also be morphologically similar: both *Amusiotheres obtusidentatus* (Dai, 1980) and *Limotheres nasutus* Holthuis, 1975 have a relatively more angular, elongated shape with a defined rostrum. A presumed morphological convergence to a rounder carapace in the outgroup species can be observed for the two bivalve‐associated species *Scleroplax faba*, and *S*. *littoralis* (Figure 5: crab C) when they are together compared with the ingroup species with the shortest morphological distance to the outgroup, *Zaops ostreum* (Say, 1817). For morphological comparison to the burrow/tube‐dwelling outgroup species *Glassella floridana* (Rathbun, 1918), see above (Figure 3).

The three above‐mentioned convergence events were statistically tested for their significance (Table 2), indicated by their similarity‐based measures (C‐values) and significance.

**TABLE 2:** Similarity‐based measures of converge for three presumed convergence events in pea crab species combinations (1000 replicates, PC1 to PC3; 84.5% of the data explained)

Species combinations		C_1_	C_2_	C_3_	C_4_	*p*‐Value for C_1_
(I)	*Dissodactylus crinitichelis*, *Dissodactylus latus*	0.816	0.299	0.525	0.952	**.004**
(II)	*Solenotheres prolixus*, *Juxtafabia muliniarum*, *Austinotheres angelicus*	0.845	0.139	0.500	0.031	**<.001**
(III)	*Scleroplax faba*, *Scleroplax littoralis*, *Zaops ostreum*	0.657	0.221	0.391	0.049	**<.001**

*Note*: Due to *Zaops ostreum* (Say, 1817) being the ingroup species with the shortest overall distance to the two *Scleroplax* species, this species was chosen to represent the ingroup in this (III) calculation. *p*‐Values indicating the probability that the degree of convergence exceeds what would be expected from a randomly evolving lineage are in **bold** if significant (*p* < .05).

A PGLS analysis was performed to test the dependency of the morphometric data, taking the phylogenetic history of the included species into account. The PGLS resulted in an insignificant *p*‐value (*p* = .823; *R* ^2^ = .151). Thus, the landmark (shape) data and the placement within the morphospace of the 33 included species are not associated with a host group, once phylogenetic nonindependence is taken into consideration (Figure 5). The comparative Procrustes ANOVA test, excluding the phylogenetic framework, resulted in a lower but also insignificant *p*‐value (*p* = .285; *R* ^2^ = .247).

## 4 DISCUSSION AND CONCLUSIONS

### 4.1 Shape differences

Although the pairwise Procrustes ANOVA shows significant differences between the mean carapace shapes of the host‐associated groups (Table 1), the differences appear to be very inconspicuous (Figure 4). The mean shape of all bivalve‐associated pinnotherine species included in this study was very similar to that of the entire set of the analyzed ingroup species. Therefore, assigning a particular pea crab carapace shape to a specific host group seems impossible at this point. However, hypotheses can be made to explain the observed shape variation of the data, based on possible physical limitations of the habitat. For instance: ascidian‐associated species might need a more elongated shape (Figure 4) to fit in the basket‐like structure inside the body of the host, and the limited space of the mantle tissue of gastropods might result in wider (and perhaps flatter) shaped associates (Figure 4) to keep the fragile appendages covered. The angular shape of echinoid ectosymbionts appears to be very typical for this host association, but all species in this group are closely related and part of the “*Dissodactylus* complex.” Members of this complex are known to have evolved a wide range of morphological adaptations on their appendages and have a flattened body shape, which might be beneficial for living an ectosymbiotic lifestyle (see de Gier & Becker, 2020). Perhaps the angularly shaped carapace margins might be linked to this dorsal flattening of the body.

Different *p*‐values are observed for the same Procrustes pairwise ANOVA tests, depending on whether the outgroup is included or not in the analyses. Although these differences might be unexpected due to most of the ingroup data being uniformly placed in both analyses (e.g., when comparing the gastropod and ectosymbiotic echinoid associates; Table 1), they can be explained by the way the *p*‐values are calculated. The *p*‐values are based on the permutation of residuals, meaning that if the morphospace is expanded or condensed, the available data for running permutations would also change (Adams & Collyer, 2018; Kaliontzopoulou, pers. comm.). This also means that choosing appropriate outgroups is extremely important in morphometric analyses.

### 4.2 Convergence events and host specificity

Hultgren et al. (2022) already suggest that two bivalve‐associated species (*Scleroplax faba* and *S*. *littoralis*; both included in the present analyses as outgroup) have evolved from having a wide carapace shape (as can be seen in the tube‐ and burrow‐dwelling outgroup species) to having a relatively round carapace. The mean AR (carapace aspect ratio) of these two species was found to be significantly higher than the pinnotherine bivalve associates, but the green‐colored branches of Figure 2 in Hultgren et al. (2022) clearly show the species to be rounder than their pinnixine relatives. Here, this proposed convergence between members of two subfamilies was shown in the phylomorphospace of the first two PCs (Figure 5, III), where the ancestral lineage of these two species shifts into the direction of the ingroup, with *S. faba* being more closely placed to the ingroup than *S. littoralis*. This difference in placement might be the result of an independent evolution of *S. faba*, evolving to be rounder, with the widest part of the carapace being on the lower side. The similarity‐based measures for convergence (C_1_ to C_4_) indicate that these taxa (*S. faba* and *S. littoralis*, compared with a member of the ingroup, *Zaops ostreum*) show an average of 65.7% convergence (Table 2; following Stayton, 2015). Although this is a rather low value due to the distance between the three species, under a Brownian motion model, this result is significant (*p* < .001), indicative of “true” convergence between these members of the in‐ and outgroup. Similarly, the two holothurian‐associated outgroup species (*Pinnixa barnharti* Rathbun, 1918 and *Pinnixa tumida* Stimpson, 1858) seem to have undergone a similar presumed convergence event, shifting away from the rest of the outgroups towards the ingroup (Figure 3). This possible convergence might be related to the endosymbiotic host choice of these two species, as is also discussed for *S. faba* and *S. littoralis* by de Gier and Becker (2020) and Hultgren et al. (2022). However, whether these two *Pinnixa* species are phylogenetically related is unknown, although *P*. *barnharti* is found in Californian waters (Zmarzly, 1992), whereas *P*. *tumida* is known from Japan and China (Dai & Yang, 1991), which might suggest they are not very closely related. DNA analyses are needed to investigate their relatedness to the other species of *Pinnixa*, and consequently if their carapace shapes are the result of a single or of two separate convergence events associated with their host choice.

Another trend seen in the data is the convergence from different lineages in the ingroup, where branches move along the morphospace towards a wider body shape (Figure 5, II). In contrast to the outgroups, the three convergence events in the branches (including the bivalve‐associated *Austinotheres angelicus*, *Juxtafabia muliniarum*, and *Solenotheres prolixus*) cannot easily be attributed to their host choice on a phylum level. While *A. angelicus* and *J. muliniarum* are relative generalists, inhabiting various families of bivalves, *S. prolixus* has been found so far exclusively in the elongated bivalve *Solen corneus* Lamarck, 1818. *Juxtafabia muliniarum* can also be found in one species of elongated bivalve, *Tagelus affinis* (Adams, 1852) (see de Gier & Becker, 2020). A potential correlation between the shape of the host bivalve and the carapace shape of the symbiont cannot be tested with the current datasets, but more symbionts of elongated bivalves can be found among the pinnotherine (some of which take wide and/or otherwise aberrant shapes: e.g., *Raytheres* (Campos, 2004), *Serenotheres* Ahyong & Ng, 2005, and *Visayeres* Ahyong & Ng, 2007(Ahyong & Ng, 2007; Campos, 2002; Ng & Meyer, 2016)). The C_1_ value of this event shows an average of 84.5% convergence, with a highly significant probability (*p* < .001; Table 2).

There are several other species with a wide carapace, of which no phylogenetic information was available (see Figure 4). For this reason, no conclusions on the origins of their wide body shape and potential convergence events can be drawn. Most notably are the holothurian‐associated *Holothuriophilus trapeziformis*, the giant‐clam‐associated *Xanthasia murigera* White, 1846, and the gastropod‐associated *Mesotheres unguifalcula* and the three species of *Orthotheres* Sakai, 1969.

An example of a convergence event where the host choice seems to have played at least a partial role can be found within the *Dissodactylus* complex (Figure 5, I). *Dissodactylus* and *Clypeasterophilus* Campos & Griffith, 1990 are two closely related paraphyletic genera, known for their ectosymbiotic association with echinoids (de Gier & Becker, 2020; Griffith, 1987; Palacios Theil et al., 2016). Two species of *Dissodactylus* were found to move in the phylomorphospace along the first PC axis in a similar manner, after splitting from their theoretical ancestor, away from the rest of the species complex: *D. crinitichelis* and *D. latus*. These species appear to have a relatively wide carapace shape, compared with the rest of the clade (Figure 5, I; see crab A). When running a similarity‐based convergence measure analysis for these two species, a C_1_ value of 81.6% was found, with a significant *p*‐value of .004 (Table 2). This relatively higher *p*‐value could be an indication of incomplete convergence or parallel shifts in the morphology (see Grossnickle et al., 2020). Both species occur in different, but overlapping regions in the tropical West Atlantic, living on various species of flattened echinoids (*D. crinitichelis* being more generalistic in its host choice; see de Gier & Becker, 2020). The current plots suggest that these species both could have occupied a similar niche in the same distribution range, and therefore evolved towards a similar shape. The sister species of *D. crinitichelis*, *Dissodactylus nitidus* Smith, 1870, has a more rounded carapace, and might be expected to live on different host groups. However, *D. nitidus* can be found on similar flattened sand dollars, albeit on different species from the East Pacific (de Gier & Becker, 2020). A detailed study focusing on the historical biogeography and morphological ancestral character states of the species in the *Dissodactylus* complex needs to be done in order to disentangle the two genera, resolve their phylogenetic positions, and discern if certain body shapes are favorable for living on specific host species. Host switching within one life history, as was shown by Jossart et al. (2013) in *Dissodactylus primitivus* Bouvier, 1917, might also play an important role in the morphological plasticity of the genera. In addition, 3D morphometrics might be applied to also acquire information about their flattened body shapes, which might be linked to the widened carapace shapes and their host choices, also suggested for other genera by Hultgren et al. (2022).

The PGLS analysis, evaluating whether the host associations explain the variation of shape, given the phylogenetic framework of the 33 included species, resulted in an insignificant *p*‐value. This means that the shape differences are not associated with the host groups, once phylogenetic nonindependence is taken into account. The linear model, excluding the phylogenetic framework, also resulted in an insignificant *p*‐value. While this means that the shape differences between these 33 included species are not explained by the predefined host associations, performing the analysis on the full dataset (181 specimens) results in contrasting, significant values (*p* = .001, *R* ^2^ = .347). The low sample size of the previous tests (18.8% of the original data) might be the case of the deviating results of the analyses (see Mundry, 2014). Increasing the sample size by including molecular data of more pea crab species might prove if the phylogenetic framework plays a significant role in the diversification of the entire (sub)family.

### 4.3 Ancestral reconstructions and ecomorphological trends

Besides analyzing convergence events, the phylomorphospace approach allows for a close examination of the evolution of shape in the deeper branches of the phylogeny (e.g., Ford et al., 2016). The currently presented phylogeny reconstruction “starts” with a somewhat widened outgroup species, *Tetrias fischerii*, and the much‐more widened species *Parapinnixa cortesi* (Figure 5). This first bivalve‐associated species is plotted between the large cloud of ingroup species, and the main tube‐ and burrow‐dwelling outgroups (including its currently designated sister species *P. cortesi*).

In the phylogeny reconstructions of Palacios Theil et al. (2016), the placement of *Parapinnixa* and *Tetrias* Rathbun, 1898, as well as *Sakaina* and *Pseudopinnixa* Ortmann, 1894, appears to be unstable due to low support values. In the phylogeny reconstruction resulting from a concatenated alignment, Palacios Theil et al. (2016; fig. 7) found a clade combining *Tetrias* and *Pseudopinnixa* to have the most basal placement, after which a clade including *Sakaina* and *Parapinnixa* branch off. In another study, these clades switch places, with high support values for the basal placement of *Parapinnixa* and *Sakaina* (Palacios Theil & Felder, 2020a; Figure 2). In addition, the placement of the Pinnixulalinae appears to be switching from a placement next to the Pinnotherinae (Palacios Theil et al., 2016), to a placement basal to the other two subfamilies (Palacios Theil & Felder, 2020a). Taking the wide body shapes of the Pinnixulalinae into account, it is likely that the typical round body shapes of symbiotic pinnotherine pea crabs have here evolved once, and that the carapace of the ancestor of the entire clade including the three subfamilies was broadly shaped. In addition, the somewhat round body shape of *Tetrias* could have evolved independently to live within a host. Future phylogenetic studies, including more species, will probably result in different tree topologies and new lineages, and will consequently be able to test hypotheses about the ancestral morphology of the most recent ancestor of the entire family (including *Tetrias*, *Parapinnixa*, and other related pea crab genera without a stable subfamily status).

### 4.4 Future perspectives

A problem posed by the currently presented data was a large number of species with unknown host, presumed free‐living, or with generalist host associations. Not all of these species will truly be free‐living and might have been dislodged or wandering from their host organism when sampled (McDermott, 2009). Using the present morphospace plots, the association type (endo‐ or ectosymbiotic) might be speculated by looking at the data clouds (e.g., the outgroup species *Pinnixulala heardi* Felder & Palacios Theil, 2020b, whose carapace shape is perfectly in line with the other tube‐ and burrow‐dwelling outgroups, as was already speculated by Felder and Palacios Theil (2020b); Figure 3). Within the ingroup, “predicting” a specific host type for species without a known association may be more difficult, and unexpected host associations might influence the shape of the convex hulls in the morphospaces and consequently influence the *p*‐values of the pairwise (M)ANOVA (Table 1). Similarly, landmarking extremes in the morphological variation of certain (often synonymized) species (e.g., within *Arcotheres*; Ng & Ahyong, 2022) might result in unexpected shifts in the datapoint clouds. In future studies the number of specimens per species should be increased, to account for this variation. In addition, efforts should be made to make the dataset as complete as possible by adding host records of species that currently have an unknown host association, or have been regarded as free‐living as for now. Moreover, a large number of species that could not be included in this study due to the lack of images might be added in the future. In the case of convergence analyses, additional molecular data are needed to include more species in the phylogeny reconstruction, and therefore in the phylomorphospace. This way, the statistical strength of the methods used in this study can be improved considerably. These more elaborate analyses can only be done if future works in the field of pea crab systematics, phylogenetics, and ecology allow for it.

Using (phylo)morphometrics allows users to visualize morphological evolutionary pathways and to see shape variation on a scale that might not be perceivable by using measurements only (e.g., “traditional morphometrics”; Zelditch et al., 2012). The field of ecomorphology (sensu Feilich & López‐Fernández, 2019) has been using the currently presented methods for years, studying the evolution of shape as a result of ecological factors, mainly in vertebrates (Claverie & Wainwright, 2014; Curth et al., 2017; Dugo‐Cota et al., 2019; Kulemeyer et al., 2009; Sherratt et al., 2019), and less so in invertebrates (Bush et al., 2006; Malcicka et al., 2017). This is due to the sampling limitation of selecting homologous 2D, or 3D landmarks in all samples (Zelditch et al., 2012). Firm homologous structures like skeletons seem to be easier to compare, but soft‐bodied invertebrate taxa pose a problem in this respect. Invertebrates like mollusk bivalves or gastropods have, however, been studied thanks to their hard (partial) exoskeleton (Burridge et al., 2015; Potkamp et al., 2017; Sherratt et al., 2016; Verhaegen et al., 2018), but for unknown reasons, crustaceans seem to be underrepresented in the field of eco‐, and phylomorphometrics (with the exception of Marochi & Masunari, 2016; Trontelj et al., 2012; Vermeiren et al., 2021).

In the current study, the phylomorphospace approach shows already multiple presumed convergent evolutionary pathways in a limited phylogenetic framework. The presented data suggest that host‐switching events could have had an important role in the evolution of carapace shapes of non‐pinnotherinae pea crabs, moving from a tube/burrow‐dwelling biology to a strictly endosymbiotic lifestyle within bivalves, in adult crabs. Within the pinnotherinae, however, host switches between phyla seem to have had almost no effect on the evolution of carapace shape. This suggests that a shift in lifestyle from ecto‐ to endosymbiotic could be the driver for carapace (and overall body) shape diversification, rather than between‐phyla host switches. This might also be the case in other symbiotic crustacean taxa with similar evolutionary switches in their lifestyles. For example, various palaemonid shrimp lineages have evolved from having a free‐living lifestyle to a life in symbiosis with an invertebrate host (e.g., Frolová et al., 2022). In addition, some lineages have had multiple between‐phyla host switches, some resulting in a shift from ecto‐ to endosymbiosis (Chow et al., 2021; Horká et al., 2016). These evolutionary pathways resulted in a wide range of morphological adaptations, ranging from changes in the morphology of the walking legs, eyes, and overall carapace shape (Dobson et al., 2014; Fransen, 1994, 2002). Although not studied in detail, symbiotic amphipods from the family Leucothoidae (inhabiting coral rubble, but also bivalve, ascidian, and sponge hosts) might also have diversified in a similar matter (e.g., White, 2011). Lastly, the extremely specious copepod order Harpacticoida has had multiple shifts from a free‐living life to a commensal or parasitic ecto‐ or endosymbiosis. A wide range of vertebrate and invertebrate hosts are utilized by these copepods, which is possibly the driver for their body shape diversification (e.g., Huys, 2016).

## AUTHOR CONTRIBUTIONS

**Werner de Gier:** Conceptualization (lead); data curation (lead); formal analysis (lead); investigation (lead); methodology (lead); project administration (lead); resources (lead); visualization (lead); writing – original draft (lead); writing – review and editing (lead).

## FUNDING INFORMATION

This project was funded by Naturalis Biodiversity Center (Leiden, The Netherlands).

## Supporting information


Supinfo

## Data Availability

The data that supports the findings of this study are available in the supplementary material of this article.
