# Supplementary material for: Phylomorphometrics reveal ecomorphological convergence in pea crab carapace shapes (Brachyura, Pinnotheridae)
Source: Ecol Evol. 2023 Jan 16;13(1):e9744. doi: 10.1002/ece3.9744 (PMC9842789; doi:10.1002/ece3.9744)

**Electronic Supplementary Material**

**Phylomorphometrics reveal ecomorphological convergence in pea crab carapace shapes (Brachyura, Pinnotheridae)**

**Werner de Gier^1,2^** (ORCID: 0000-0002-2137-2052)

^1^: Naturalis Biodiversity Center, Darwinweg 2, 2333 CR Leiden, The Netherlands

^2^: Groningen Institute for Evolutionary Life Sciences, University of Groningen, P.O. Box 11103, 9700 CC Groningen, The Netherlands

werner.degier@naturalis.nl

**Appendix S1.** Species with figures available in literature but excluded from the currently presented analyses due to there not being usable figures for these purposes (criteria for figure selection can be found in the material and methods section), with their presumed host association. Unknown host associations are indicated with a question mark (?).

| **Genus** | **Species** | **Author(s)** | **Host phylum** |
| --- | --- | --- | --- |
| *Abyssotheres* | *A.* *abyssicola* | (Alcock & Anderson, 1899) | Bivalves |
| *Afropinnotheres* | *A.* *guinotae* | Manning, 1993c | Bivalves |
| *Alainotheres* | *A.* *leloeuffi* | (Crosnier, 1969) | ? |
| *Amusiotheres* | *A.* *hanumantharaoi* | Devi & Shyamasundari, 1989 | Bivalves |
| *Arcotheres* | *A.* *atrinae* | (Sakai, 1939) | Bivalves |
|  | *A.* *lanensis* | (Rathbun, 1909) | ? |
| *Enigmatheres* | *E. canfieldi* | (Rathbun, 1918) | Gastropods |
| *Fabia* | *F. concharum* | (Rathbun, 1894) | Bivalves |
|  | *F. felderi* | Gore, 1986 | ? |
|  | *F. malaguena* | (Garth, 1948) | ? |
| *Nannotheres* | *N. moorei* | Manning & Felder, 1996 | Bivalves |
| *Nepinnotheres* | *N. fulvia* | Ahyong & Ng, 2020 | Bivalves |
|  | *N. glaberrimus* | (Bürger, 1895) | Bivalves |
|  | *N. margaritiferae* | (Laurie, 1906) | Bivalves |
|  | *N. rouxi* | (H. Milne Edwards, 1853) | ? |
| *Pinnaxodes* | *P. bipunctatus* | (Nicolet, 1849) | Echinoids (?) |
| *Pinnotheres* | *P. bidentatus* | Sakai, 1939 | ? |
|  | *P. coutierei* | Nobili, 1905 | ? |
|  | *P. gordoni / gordonae* | Shen, 1932 | Bivalves |
|  | *P. guerini* | H. Milne Edwards, 1853 | Bivalves |
|  | *P. hirtimanus* | H. Milne Edwards, 1853 | ? |
|  | *P. kutensis* | Rathbun, 1909 | ? |
|  | *P. lithodomi* | Smith, 1870 | Bivalves |
|  | *P. lutescens* | Nobili, 1905 | ? |
|  | *P. matricola* | Alcock, 1900 | Bivalves |
|  | *P. obesus* | Dana, 1852 | Bivalves |
|  | *P. obscurus* | Stimpson, 1858 | ? |
|  | *P. perezi* | Nobili, 1905 | Bivalves |
|  | *P. pichilinquei* | Rathbun, 1923 | ? |
|  | *P. siamensis* | Rathbun, 1909 | ? |
|  | *P. trichopus* | Tesch, 1918 | Bivalves |
| *Viridotheres* | *V. buergeri* | (Rathbun, 1909) | ? |
|  | *V. kupang* | Ahyong, 2018 | ? |
|  | *V. sanguinolariae* | (Pillai, 1951) | Bivalves |
| *Zaops* | *Z. geddesi* | (Miers, 1880) | Bivalves |

**Appendix S2.** All included species (including outgroup species), and the sources for their used figures. For references, see below. *: Newly obtained photos, see main article (fig. 1). **: Newly obtained, unpublished, microCT scans. ‘Nr.’ Indicates the number of the specimen in Appendix S3.

| **Genus** | **Species** | **Author(s)** | **Nr.** | **Figure source** | **Host group** | **Figure** |
| --- | --- | --- | --- | --- | --- | --- |
| **INGROUPS** | | | | | | |
| *Abyssotheres* | *A. acesticola* | Komatsu & Ohtsuka, 2009 | 1 | Komatsu & Ohtsuka, 2009 | Bivalves | Fig. 1A |
| *Afropinnotheres* | *A. crosnieri* | Manning, 1993 | 2 | Manning, 1993c | Bivalves | Fig. 3a |
|  | *A. dofleini* | Lenz (in Lenz & Strunck, 1914) | 3 | Ng, 2018 | Generalist | Fig. 7A |
|  | *A. larissae* | (Machkevskiy, 1992) | 4 | Manning, 1993c | Bivalves | Fig. 7b |
|  | *A. monodi* | Manning, 1993 | 5 | Manning, 1993c | Bivalves | Fig. 10a |
|  | *A. ratnakara* | Ng & Kumar, 2015 | 6 | Ng & Kumar, 2015 | Bivalves | Fig. 2A |
| *Alain* | *A. crosnieri* | Manning, 1998 | 7 | Manning, 1998 | Holothurians (Endo-) | Fig. 1B |
|  | *A. raymondi* | Ahyong & Ng, 2008 | 8 | Ahyong & Ng, 2008 | Holothurians (Endo-) | Fig. 2A |
| *Amusiotheres* | *A. obtusidentatus* | (Dai, Feng, Song & Chen, 1980) | 9 | Ng & Ho, 2016a | Bivalves | Fig. 2B |
| *Arcotheres* | *A. alcocki* | (Rathbun, 1909) | 10 | Ng & Ahyong, 2022 | Bivalves | Fig. 54A |
|  | *A. boninensis* | (Stimpson, 1858) | 11 | Ahyong & Ng, 2021 | Bivalves | Fig. 9A |
|  | *A. cyclinus* | (Shen, 1932) | 12 | Dai & Yang, 1991 | Bivalves | Fig. 207.1 |
|  | *A. excussus* | (Dai, Feng, Song & Chen, 1980) | 13 | Ng & Ngo, 2022 | Bivalves | Fig. 4A |
|  | *A. exiguus* | (Bürger, 1895) | 14 | Ng & Ahyong, 2022 | Unknown | Fig. 35B |
|  | *A. latifrons* | (Bürger, 1895) | 15 | Ahyong & Ng, 2007a | Unknown | Fig. 5A |
|  | *A. nudifrons* | (Bürger, 1895) | 16 | Ahyong & Ng, 2007a | Unknown | Fig. 8A |
|  | *A. obesus* | (Dana, 1852) | 17 | Ng & Ahyong, 2022 | Bivalves | Fig. 55A |
|  | *A. ocularius* | Komai, Kawai & Ng, 2020 | 18 | Ng & Ahyong, 2022 | Bivalves | Fig. 23A |
|  | *A. palaensis* | (Bürger, 1895) | 19 | Ng & Ahyong, 2022 | Bivalves | Fig. 12C |
|  | *A. pernicola* | (Bürger, 1895) | 20 | Ahyong & Ng, 2007a | Bivalves | Fig. 10F |
|  | *A. placunae* | (Hornell & Southwell, 1909) | 21 | Trivedi et al, 2018 | Bivalves | Fig. 1C |
|  | *A. placunicola* | Ng, 2018 | 22 | Ng & Ahyong, 2022 | Bivalves | Fig. 58A |
|  | *A. pollus* | Ahyong & Ng, 2020 | 23 | Ahyong & Ng, 2020 | Bivalves | Fig. 2A |
|  | *A. purpureus* | (Alcock, 1900) | 24 | Ahyong & Ng, 2021 | Bivalves | Fig. 12A |
|  | *A. quadratus* | (Rathbun, 1909) | 133 | * | Bivalves | Fig. 1C |
|  | *A. rayi* | Ahyong & Ng, 2007 | 25 | Ng & Ahyong, 2022 | Bivalves | Fig. 48B |
|  | *A. rotundatus* | (Bürger, 1895) | 26 | Ahyong & Ng, 2007a | Bivalves | Fig. 13A |
|  | *A. shahi* | Trivedi, Campos & Vachhrajani, 2018 | 27 | Trivedi et al., 2018 | Bivalves | Fig. 1A |
|  | *A. similis* | (Bürger, 1895) | 28 | Ng & Ahyong, 2022 | Bivalves | Fig. 28A |
|  | *A. sinensis* | (Shen, 1932) | 29 | Dai & Yang, 1991 | Bivalves | Fig. 206.1 |
|  | *A. tivelae* | (Gordon, 1936) | 30 | Ng, Clark & Naderloo, 2022 | Bivalves | Fig. 1D |
| *Austinotheres* | *A. angelicus* | (Lockington, 1877) | 31 | Campos, 2002 | Bivalves | Fig. 1A |
| *Austrotheres* | *A. holothuriensis* | (Baker, 1907) | 32 | Ahyong, 2018 | Generalist | Fig. 7A |
|  | *A. pregenzeri* | Ahyong, 2018 | 33 | Ahyong, 2018 | Ascidians | Fig. 6A |
| *Bonita* | *B. mexicana* | Campos 2009 | 34 | Campos, 2009 | Bivalves | Fig. 2B |
| *Buergeres* | *B. choprai* | Ahyong & Ng, 2020 | 35 | Ahyong & Ng, 2020 | Holothurians (Endo-) | Fig. 3A |
|  | *B. deccanensis* | (Chopra, 1931) | 36 | Chopra, 1931 | Holothurians (Endo-) | Fig. 4 (pl. 7) |
|  | *B. holothuriae* | (Semper, 1880) | 37 | Ahyong & Ng, 2007a | Holothurians (Endo-) | Fig. 15A |
|  | *B. ortmanni* | (Bürger, 1895) | 38 | Ahyong & Ng, 2007a | Holothurians (Endo-) | Fig. 16A |
| *Calyptraeotheres* | *C. camposi* | Ayón-Parente & Hendrickx, 2014 | 39 | Ayón-Parente & Hendrickx, 2014 | Gastropods | Fig. 1A |
|  | *C. garthi* | (Fenucci, 1975) | 40 | Campos, 1999 | Gastropods | Fig. 1E |
|  | *C. granti* | (Glassell, 1933) | 41 | Campos, 1990 | Gastropods | Fig. 1A |
|  | *C. hernandezi* | Hernández-Ávila & Campos, 2006 | 42 | Hernández-Ávila & Campos, 2006 | Gastropods | Fig. 1a |
|  | *C. pepeluisi* | Campos & Hernández-Ávila, 2010 | 43 | Campos & Hernández-Ávila, 2010 | Unknown | Fig. 3A |
|  | *C. politus* | (Smith, 1870) | 44 | Campos, 1999 | Gastropods | Fig. 1C |
| *Clypeasterophilus* | *C. juvenilis* | (Bouvier, 1917) | 45 | ** | Echinoids (Ecto-) | N.A. |
|  | *C. rugatus* | (Bouvier, 1917) | 46 | ** | Echinoids (Ecto-) | N.A. |
|  | *C. stebbingi* | (Rathbun, 1918) | 47 | ** | Echinoids (Ecto-) | N.A. |
|  | *C. ususfructus* | (Griffith, 1987) | 48 | Griffith, 1987 | Echinoids (Ecto-) | Fig. 3A |
| *Discorsotheres* | *D. camposi* | Ahyong, 2018 | 49 | Ahyong, 2018 | Bivalves | Fig. 10A |
|  | *D. spondyli* | (Nobili, 1905) | 50 | Ahyong, 2018 | Bivalves | Fig. 11A |
|  | *D. subglobosus* | (Baker, 1907) | 51 | Ahyong, 2018 | Bivalves | Fig. 9A |
|  | *D. subquadratus* | (T. Sakai, 1939) | 52 | Ahyong, 2018 | Bivalves | Fig. 12A |
| *Dissodactylus* | *D. crinitichelis* | Moreira, 1901 | 53 | ** | Echinoids (Ecto-) | N.A. |
|  | *D. glasselli* | Rioja, 1944 | 54 | Griffith, 1987 | Echinoids (Ecto-) | Fig. 9H |
|  | *D. latus* | H. Griffith, 1987 | 55 | Griffith, 1987 | Echinoids (Ecto-) | Fig. 5A |
|  | *D. lockingtoni* | Glassell, 1935 | 56 | Griffith, 1987 | Echinoids (Ecto-) | Fig. 9B |
|  | *D. mellitae* | (Rathbun, 1900) | 57 | Griffith, 1987 | Echinoids (Ecto-) | Fig. 9I |
|  | *D. nitidus* | Smith, 1870 | 58 | Griffith, 1987 | Echinoids (Ecto-) | Fig. 9A |
|  | *D. primitivus* | Bouvier, 1917 | 59 | ** | Echinoids (Ecto-) | N.A. |
|  | *D. schmitti* | H. Griffith, 1987 | 60 | Griffith, 1987 | Unknown | Fig. 4A |
|  | *D. xantusi* | Glassell, 1936 | 61 | Griffith, 1987 | Echinoids (Ecto-) | Fig. 9C |
| *Durckheimia* | *D. caeca* | Bürger, 1895 | 62 | Ahyong & Ng, 2007a | Bivalves | Fig. 17A |
|  | *D. carnipes* | De Man, 1889 | 63 | Ahyong & Ng, 2005 | Unknown | Fig. 1A |
|  | *D. lochi* | Ahyong & Brown, 2003 | 64 | Ahyong & Brown, 2003 | Bivalves | Fig. 1A |
| *Ernestotheres* | *E. conicola* | Manning, 1993 | 65 | Manning, 1993c | Gastropods | Fig. 13b |
| *Fabia* | *F. byssomiae* | (Say, 1818) | 66 | Campos, 1996b | Bivalves | Fig. 1A |
|  | *F. carvachoi* | Campos, 1996 | 67 | Campos, 1996b | Bivalves | Fig. 2A |
|  | *F. emiliae* | (Melo, 1971) | 68 | Campos, 2013 | Bivalves | Fig. 3A |
|  | *F. hemphilli* | (Rathbun, 1918) | 69 | Campos, 2013 | Unknown | Fig. 2A |
|  | *F. subquadrata* | Dana, 1851 | 70 | Campos, 1996b | Bivalves | Fig. 6A |
|  | *F. tellinae* | Cobb, 1973 | 71 | Campos, 1996b | Bivalves | Fig. 8A |
| *Gemmotheres* | *G. chamae* | Roberts, 1975 | 72 | Campos & Hernández-Aguilera, 2020 | Bivalves | Fig. 1A |
| *Holotheres* | *H. danielae* | Ahyong, 2010 | 73 | Ahyong, 2010 | Holothurians (Endo-) | Fig. 1A |
|  | *H. flavus* | (Nauck, 1880) | 74 | Ng & Manning, 2003 | Holothurians (Endo-) | Fig. 3A |
|  | *H. halingi* | (Hamel, Ng & Mercier, 1999) | 75 | Hamel et al., 1999 | Holothurians (Endo-) | Fig. 2A |
|  | *H. semperi* | (Bürger, 1895) | 76 | Ng & Manning, 2003 | Holothurians (Endo-) | Fig. 1J |
|  | *H. setnai* | (Chopra, 1931) | 77 | Chopra, 1931 | Holothurians (Endo-) | Fig. 3 (pl. 7) |
| *Holothuriophilus* | *H. pacificus* | (Poeppig, 1836) | 78 | Manning 1993b | Holothurians (Endo-) | Fig. 2a |
|  | *H. trapeziformis* | Nauck, 1880 | 79 | Campos et al, 2012 | Holothurians (Endo-) | Fig. 2A |
| *Hospitotheres* | *H. powelli* | Manning, 1993 | 80 | Manning, 1993c | Unknown | Fig. 16a |
| *Juxtafabia* | *J. muliniarum* | (Rathbun, 1918) | 81 | Campos, 1993 | Bivalves | Fig. 6 |
| *Latatheres* | *L. affinis* | (H. Milne Edwards, 1853) | 82 | Ahyong, 2018 | Unknown | Fig. 15A |
|  | *L. tomentipes* | (Takeda & Konichi, 1994) | 83 | Ahyong, 2018 | Bivalves | Fig. 14A |
| *Limotheres* | *L. nasutus* | Holthuis, 1975 | 84 | Holthuis, 1975 | Bivalves | Fig. 1a |
| *Magnotheres* | *M. globosus* | (Hombron & Jacquinot, 1846) | 85 | Ng & Ahyong, 2022 | Bivalves | Fig. 74A |
| *Mesotheres* | *M. barbatus* | (Desbonne, in Desbonne & Schramm, 1867) | 86 | Ng et al, 2019 | Gastropods | Fig. 7A |
|  | *M. serrei* | (Rathbun, 1909) | 87 | * | Gastropods | Fig. 1B |
|  | *M. strombi* | (Rathbun, 1905) | 88 | Ng et al, 2019 | Gastropods | Fig. 4A |
|  | *M. unguifalcula* | (Glassel, 1936) | 89 | Campos, 1989a | Gastropods | Fig. 2a |
| *Nepinnotheres* | *N. affinis* | (Bürger, 1895) | 91 | Ahyong & Ng, 2007a | Bivalves | Fig. 21A |
|  | *N. africanus* | Manning, 1993 | 92 | Manning, 1993c | Unknown | Fig. 18a |
|  | *N. androgynus* | Manning, 1993 | 93 | Manning, 1993c | Bivalves | Fig. 20b |
|  | *N. atrinicola* | (Page, 1983) | 94 | Page, 1983 | Bivalves | Fig. 3H |
|  | *N. cardii* | (Bürger, 1895) | 95 | Ahyong & Ng, 2007a | Bivalves | Fig. 22A |
|  | *N. corbiculae* | (T. Sakai, 1939) | 96 | Sakai, 1976 | Bivalves | Fig. 4 (pl. 200) |
|  | *N. edwardsi* | (De Man, 1887) | 97 | De Man, 1887 | Bivalves | Fig. 6 (pl. 6) |
|  | *N. latipes* | (Jacquinot in Hombron & Jacquinot, 1846) | 98 | Ahyong, 2020b | Bivalves | Fig. 2A |
|  | *N. novaezelandiae* | (Filhol, 1885) | 99 | Page, 1983 | Bivalves | Fig. 1H |
|  | *N. pectinicola* | (Bürger, 1895) | 100 | Ahyong & Ng, 2007a | Bivalves | Fig. 3C |
|  | *N. pinnotheres* | (Linnaeus, 1758) | 101 | Becker & Turkay, 2010 | Generalist | Fig. 3A |
|  | *N. rathbunae* | (Schmitt, McCain & Davidson, 1973) | 102 | Ahyong & Ng, 2007a | Bivalves | Fig. 3D |
|  | *N. sanqueri* | Manning, 1993 | 103 | Manning, 1993c | Unknown | Fig. 26a |
|  | *N. tellinae* | (Manning & Holthuis, 1981) | 104 | Manning, 1993c | Bivalves | Fig. 28a |
|  | *N. tsingtaoensis* | (Shen, 1932) | 105 | Dai & Yang, 1991 | Bivalves | Fig. 217.1 |
|  | *N. villosulus* | (Guérin, 1832) | 106 | Miers, 1886 | Bivalves | Fig. 2a (pl. 22) |
| *Opisthopus* | *O. transversus* | Rathbun, 1894 | 107 | Campos & Manning, 2000 | Generalist | Fig. 2C |
| *Orthotheres* | *O. baoyu* | Ng & Ho, 2016 | 108 | Ng & Ho, 2016b | Gastropods | Fig. 8A |
|  | *O. haliotidis* | Geiger & Martin, 1999 | 109 | Ng & Ho, 2016b | Gastropods | Fig. 5A |
|  | *O. turboe* | T. Sakai, 1969 | 110 | Ng & Ho, 2016b | Gastropods | Fig. 2A |
| *Ostracotheres* | *O. cynthiae* | Nobili, 1906 | 111 | Ahyong, 2018 | Ascidians | Fig. 4A |
|  | *O. tridacnae* | (Rüppell, 1830) | 112 | Ahyong, 2018 | Bivalves | Fig. 2A |
| *Pinnaxodes* | *P. chilensis* | (H. Milne Edwards, 1837) | 113 | Takeda & Masahito, 2000 | Echinoids (Endo-) | Fig. 1A |
|  | *P. floridensis* | H. W. Wells & M. J. Wells, 1961 | 114 | Wells & Wells, 1961 | Holothurians (Endo-) | Fig. 1B |
|  | *P. gigas* | Green, 1992 | 115 | Campos, 2016 | Bivalves | Fig. 3A |
|  | *P. major* | Ortmann, 1894 | 116 | Ng & Manning, 2003 | Generalist | Fig. 6A |
|  | *P. mutuensis* | (T. Sakai, 1939) | 117 | Marin, 2014 | Bivalves | Fig. 1a |
|  | *P. tomentosus* | (Ortmann, 1894) | 118 | De Melo & Boehs, 2003 | Bivalves | Fig. 3 |
| *Pinnotheres* | *P. bicristatus* | Cuesta, Garcia Raso, Abelló, Marco-Herrero, Silva & Drake, 2019 | 119 | Cuesta et al., 2019 | Bivalves | Fig. 7B |
|  | *P. dilatatus* | Shen, 1932 | 120 | Dai & Yang, 1991 | Bivalves | Fig. 216.1 |
|  | *P. globosus* | Hombron & Jacquinot, 1846 | 121 | Jacquinot in Hombron & Jacquinot, 1846 | Bivalves | Fig. 21 (pl. 5) |
|  | *P. haiyangensis* | Shen, 1932 | 122 | Dai & Yang, 1991 | Bivalves | Fig. 214.1 |
|  | *P. hickmani* | (Guiler, 1950) | 123 | Pregenzer, 1979 | Bivalves | Fig. 1.2 |
|  | *P. laquei* | T. Sakai, 1961 | 124 | Feldmann et al, 1996 | Brachiopods | Fig. 2.3 |
|  | *P. luminatus* | Dai, Feng, Song & Chen, 1980 | 125 | Dai & Yang, 1991 | Bivalves | Fig. 218.1 |
|  | *P. maindroni* | Nobili, 1905 | 126 | Nobili, 1906 | Unknown | Fig. 8 (pl. 8) |
|  | *P. onychodactylus* | Tesch, 1918 | 127 | Tesch, 1918 | Unknown | Fig. 5 (pl. 17) |
|  | *P. parvulus* | Stimpson, 1858 | 128 | Sakai, 1939 | Bivalves | Fig. 72a |
|  | *P. pectunculi* | Hesse, 1872 | 129 | * | Bivalves | Fig. 1B |
|  | *P. pholadis* | De Haan, 1835 | 130 | Dai & Yang, 1991 | Bivalves | Fig. 213.1 |
|  | *P. pilulus* | Dai, Feng, Song & Chen, 1980 | 131 | Dai & Yang, 1991 | Bivalves | Fig. 215.1 |
|  | *P. pisum* | (Linnaeus, 1767) | 132 | Becker & Turkay, 2010 | Bivalves | Fig. 5A |
|  | *P. pugettensis* | Holmes, 1900 | 133 | Rathbun, 1918 | Ascidians | Fig. 8 (pl. 17) |
|  | *P. sebastianensis* | (Rodrigues da Costa, 1970) | 134 | Rodrigues da Costa, 1970 | Echinoids (Endo-) | Fig. 2 |
|  | *P. serrignathus* | Shen, 1932 | 135 | Shen, 1932 | Unknown | Fig. 86 |
|  | *P. shoemakeri* | Rathbun, 1918 | 136 | Rathbun, 1918 | Unknown | Fig. 1 (pl. 22) |
|  | *P. taichungae* | K. Sakai, 2000 | 138 | Hsueh & Huang, 1996 | Unknown | Fig. 1A |
|  | *P. taylori* | Rathbun, 1918 | 139 | Rathbun, 1918 | Ascidians | Fig. 8 (pl. 21) |
| *Plenotheres* | *P. coarctatus* | (Bürger, 1895) | 140 | Ng & Ahyong, 2022 | Bivalves | Fig. 63D |
| *Raytheres* | *R. clavapedatus* | (Glassell, 1935) | 141 | Campos, 2002 | Bivalves | Fig. 5A |
| *Serenotheres* | *S. besutensis* | (Serène, 1967) | 142 | Ahyong & Ng 2005 | Bivalves | Fig. 5A |
|  | *S. janus* | Ng & Meyer, 2016 | 143 | Ng & Meyer, 2016 | Bivalves | Fig. 2B |
| *Sindheres* | *S. karachiensis* | Kazmi & Manning, 2003 | 144 | Kazmi & Manning, 2003 | Bivalves | Fig. 1a |
| *Solenotheres* | *S. prolixus* | Ng & Ngo, 2010 | 145 | Ng & Ngo, 2010 | Bivalves | Fig. 2A |
| *Tacitotheres* | *T. glaber* | (Bürger, 1895) | 146 | Ng et al, 2019 | Bivalves | Fig. 9B |
|  | *T. laevis* | (Bürger, 1895) | 147 | Ahyong & Ng, 2007a | Bivalves | Fig. 3E |
|  | *T. longipes* | (Bürger, 1895) | 148 | Ahyong & Ng, 2007a | Unknown | Fig. 3F |
| *Trichobezoares* | *T. pilumnoides* | (Nobili, 1906) | 150 | Ng, 2018 | Holothurians (Endo-) | Fig. 5A |
|  | *T. villosissimus* | (Doflein, 1904) | 151 | Ng, 2018 | Holothurians (Endo-) | Fig. 2A |
| *Tridacnatheres* | *T. whitei* | (de Man, 1888) | 152 | Ahyong & Ng 2005 | Bivalves | Fig. 6A |
| *Tumidotheres* | *T. carabiensis* | Palacios Theil & Felder, 2019 | 153 | Palacios Theil & Felder, 2019 | Bivalves | Fig. 5C |
|  | *T. maculatus* | (Say, 1818) | 154 | Palacios Theil & Felder, 2019 | Bivalves | Fig. 6A |
|  | *T. margarita* | (Verrill, 1869) | 155 | Campos, 1989b | Bivalves | Fig. 1a |
|  | *T. orcutti* | (Rathbun, 1918) | 156 | Campos & Vargas-Castillo, 2013 | Unknown | Fig. 5 (A) |
| *Tunicotheres* | *T. moseri* | (Rathbun, 1918) | 157 | Campos, 1996a | Ascidians | Fig. 3A |
| *Viridotheres* | *V. asaphis* | Ahyong, 2020 | 158 | Ahyong, 2020a | Bivalves | Fig. 2A |
|  | *V. cygnus* | Ahyong, 2020 | 159 | Ahyong, 2020b | Bivalves | Fig. 1A |
|  | *V. gracilis* | (Bürger, 1895) | 160 | Ahyong & Ng, 2007a | Bivalves | Fig. 25A |
|  | *V. lillyae* | (Manning, 1993) | 161 | Manning, 1993c | Unknown | Fig. 22a |
|  | *V. marionae* | Manning, 1996 | 162 | Manning, 1996 | Bivalves | Fig. 1a |
|  | *V. otto* | Anhyong & Ng, 2007 | 163 | Anhyong & Ng, 2007 | Unknown | Fig. 26A |
|  | *V. takedai* | Ahyong, Komai & Watanabe, 2012 | 164 | Ahyong et al., 2012 | Bivalves | Fig. 1A |
|  | *V. viridis* | (Manning, 1993) | 165 | Manning, 1993c | Unknown | Fig. 30a |
| *Visayeres* | *V. acron* | Ahyong & Ng, 2007 | 166 | Ahyong & Ng, 2007b | Bivalves | Fig. 1A |
| *Waldotheres* | *W. mccainae* | (Schmitt in Schmitt, McCain & Davidson, 1973) | 167 | Manning, 1993c | Bivalves | Fig. 31 |
| *Xanthasia* | *X. murigera* | White, 1846 | 168 | Ahyong & Ng, 2005 | Bivalves | Fig. 7A |
| *Zaops* | *Z. angelae* | Manning, 1993 | 169 | Manning, 1993a | Bivalves | Fig. 1a |
|  | *Z. ostreum* | (Say, 1817) | 170 | Manning, 1993b | Bivalves | Fig. 4 |
| **OUTGROUPS** | | | | | | |
| *Austinixa* | *A. roblesi* | Palacios Theil & Felder, 2020 | 178 | Palacios Theil & Felder, 2020 | Burrow/Tube | Fig. 9A |
| *Glassella* | *G. floridana* | (Rathbun, 1918) | 179 | Felder & Palacios Theil, 2020b | Burrow/Tube | Fig. 1A |
| *Indopinnixa* | *I. kasijani* | Rahayu & Ng, 2010 | 180 | Rahayu & Ng, 2010 | Free-living | Fig. 1B |
| *Parapinnixa* | *P. cortesi* | Thoma, Heard & Vargas, 2005 | 176 | Thoma et al., 2005 | Burrow/Tube | Fig. 1A |
| *Pinnixa* | *P. barnharti* | Rathbun, 1918 | 172 | Zmarzly, 1992 | Holothurians (Endo-) | Fig. 2 |
|  | *P. tumida* | Stimpson, 1858 | 173 | Dai & Yang, 1991 | Holothurians (Endo-) | Fig. 220.1 |
|  | *P. banzu* | Komai, Nishi & Taru, 2014 | 181 | Komai et al., 2014 | Burrow/Tube | Fig. 6A |
| *Pinnixulala* | *P. heardi* | Felder & Palacios Theil, 2020 | 183 | Felder & Palacios Theil, 2020a | Unknown | Fig. 4A |
| *Sakaina* | *S. glabra* | Jiang & Liu, 2011 | 177 | Jiang & Liu, 2011 | Free-living | Fig. 4A |
| *Scleroplax* | *S. faba* | (Dana, 1851) | 174 | Zmarzly, 1992 | Bivalves | Fig. 4B |
|  | *S. littoralis* | (Holmes, 1895) | 175 | Zmarzly, 1992 | Bivalves | Fig. 9B |
|  | *S. tubicola* | (Holmes, 1895) | 182 | Zmarzly, 1992 | Burrow/Tube | Fig. 16A |
| *Tetrias* | *T. fischerii* | (A. Milne-Edwards, 1867) | 171 | Sakai, 1976 | Bivalves | Fig. 3 (pl. 203) |

**References**

Ahyong, S. T., & Brown, D. E. (2003). Description of *Durckheimia lochi* n. sp., with an annotated check-list of Australian Pinnotheridae (Crustacea: Decapoda: Brachyura). Zootaxa, 254, 1–20.

Ahyong, S. T. (2010). *Holotheres danielae*, a new species of pinnotherid crab from the Indo-West Pacific (Decapoda, Brachyura), with a key to the genus. Crustaceana Monogr., 11, 35–40.

Ahyong, S. T. (2018). Revision of *Ostracotheres* H. Milne Edwards, 1853 (Crustacea: Brachyura: Pinnotheridae). Raffles Bull. Zool., 66, 538–571.

Ahyong, S. T. (2020a). Discovery of *Viridotheres* Manning, 1996 in the southwestern Pacific and first record of *Discorsotheres camposi* Ahyong, 2018 from New Caledonia (Crustacea: Brachyura: Pinnotheridae). Zootaxa, 4763, 429–434.

Ahyong, S. T. (2020b). Resolution of the identity of *Pinnotheres latipes* Hombron & Jacquinot, 1846 and description of a new species of *Viridotheres* Manning, 1996 (Decapoda: Brachyura: Pinnotheridae): Two symbionts of bivalve molluscs. J. Crustacean Biol., 1–8.

Ahyong, S. T., Komai, T., & Watanabe, T. (2012). First *Viridotheres* Manning, 1996, from Japan, with a key to the species (Decapoda, Brachyura, Pinnotheridae). In Studies on Eumalacostraca: A Homage to Masatsune Takeda; Komatsu, H., Okuno, J., Fukuoka, K., Eds.; Brill: Leiden, The Netherlands, pp. 35–48.

Ahyong, S. T., & Ng, P. K. L. (2005). Review of *Durckheimia* and *Xanthasia*, with descriptions of two new genera (Decapoda: Brachyura: Pinnotheridae). J. Crustacean Biol., 25, 116–129.

Ahyong, S. T., & Ng, P. K. L. (2007a). The pinnotherid type material of Semper (1880), Nauck (1880) and Bürger (1895) (Crustacea: Decapoda: Brachyura). Raffles Bull. Zool. Suppl., 191–226.

Ahyong, S. T., & Ng, P. K. L. (2007b). *Visayeres acron*, a new genus and species of pinnotherid crab (Crustacea: Decapoda: Brachyura) from the Philippines. Raffles Bull. Zool. Suppl., 16, 187–189.

Ahyong, S. T., & Ng, P. K. L. (2008). *Alain raymondi*, a new species of deepwater pinnotherid crab (Crustacea: Decapoda: Brachyura) from the Philippines, commensal with holothurians. Zootaxa, 1919, 61–68.

Ahyong, S. T., & Ng, P. K. L. (2020). New species of pinnotherid crabs from Southeast Asia and Papua New Guinea (Crustacea: Decapoda: Brachyura). Zootaxa, 4816, 333–349.

Ahyong, S. T., & Ng, P. K. L. (2021). The pinnotherid crabs from the Gulf of Siam described by Rathbun (1909) (Decapoda: Brachyura): revisited and revised. Raffles Bull. Zool., 69, 188–211.

Ayón-parente, M., & Hendrickx, M. E. (2014). *Calyptraeotheres* sp. nov. (Crustacea: Decapoda: Pinnotheridae), symbiont of the slipper shell *Crepidula striolata* Menke, 1851 (Mollusca: Gastropoda: Calyptraeidae) from the Gulf of California, Mexico. Zootaxa, 3872, 89–94.

Becker, C., & Türkay, M. (2010). Taxonomy and morphology of European pea crabs (Crustacea: Brachyura: Pinnotheridae). J. Nat. Hist., 44, 1555–1575.

Campos, E. (1989a). Comments on taxonomy of the genus *Orthotheres* Sakai, 1969 (Crustacea, Brachyura, Pinnotheridae). Bull. Mar. Sci., 44, 1123–1128.

Campos, E. (1989b). *Tumidotheres*, a new genus for (*Pinnotheres margarita*) Smith, 1869, and *Pinnotheres maculatus* Say, 1818 (Brachyura: Pinnotheridae). J. Crustacean Biol., 9, 672–679.

Campos, E. (1990). *Calyptraeotheres*, a new genus of Pinnotheridae for the limpet crab *Fabia granti* Glassell, 1933 (Crustacea, Brachyura). Proc. Biol. Soc. Wash., 103, 364–371.

Campos, E. (1993). Systematics and taxonomic remarks on *Pinnotheres muliniarium* Rathbun, 1918 (Crustacea: Brachyura: Pinnotheridae). Proc. Biol. Soc. Wash., 106, 92–101.

Campos, E. (1996a). Partial revision of pinnotherid crab genera with a two-segmented palp on the third maxilliped (Decapoda: Brachyura). J. Crustacean Biol., 16, 556–563.

Campos, E. (1996b). Partial revision of the genus *Fabia* Dana, 1851 (Crustacea: Brachyura: Pinnotheridae). J. Nat. Hist., 30, 1157–1178.

Campos, E. (1999). Inclusion of the austral species *Pinnotheres* *politus* (Smith, 1869) and *Pinnotheres garthi* Fenucci, 1975 within the genus *Calyptraeotheres* Campos, 1990 (Crustacea: Brachyura: Pinnotheridae). Proc. Biol. Soc. Wash., 112, 536–540.

Campos, E. (2002). Two new genera of pinnotherid crabs from the tropical Eastern Pacific (Decapoda: Brachyura: Pinnotheridae). J. Crustacean Biol., 22, 328–336.

Campos, E. (2009). A new species and two new genera of pinnotherid crabs from the northeastern Pacific Ocean, with a reappraisal of the subfamily Pinnotherinae de Haan, 1833 (Crustacea: Brachyura: Pinnotheridae). Zootaxa, 2022, 29–44.

Campos, E. (2013). Remarks on the sexual dimorphism and taxonomy of *Fabia* Dana, 1851 (Crustacea, Brachyura, Pinnotheridae). Zootaxa, 3616, 190–200.

Campos, E. (2016). The Pinnotheridae of the northeastern Pacific (Alaska to Mexico): Zoogeographical remarks and new bivalve hosts (Crustacea, Brachyura, Pinnotheridae). Zootaxa, 4170, 311–329.

Campos, E., & Hernández-Aguilera, J. L. (2020). First record and range extension of the Jewel Box clam crab *Gemmotheres chamae* (Roberts, 1975) to the Gulf of Mexico, with comments on the systematics of the pinnotherines with a 2-segmented palp on the third maxilliped (Crustacea: Brachyura: Pinnotheridae. Naupl. - J. Braz. Crustacean Soc., 28, 1–7.

Campos, E., & Hernández-ávila, I. (2010). Phylogeny of *Calyptraeotheres* Campos, 1990 (Crustacea, Decapoda, Brachyura, Pinnotheridae) with the description of *C. pepeluisi* new species from the tropical Mexican Pacific. Zootaxa, 2691, 41–52.

Campos, E., & Manning, R. B. (2000). The Identities of *Pinnotheres nudus* Holmes, 1895 and *P. nudus* sensu Weymouth, 1910 (Crustacea: Decapoda: Pinnotheridae). Proc. Biol. Soc. Wash., 113, 799–805.

Campos, E., Peláez-zárate, V. A., & Solís-marín, F. A. (2012). Rediscovery, hosts and systematics of *Holothuriophilus trapeziformis* Nauck, 1880 (Crustacea, Brachyura, Pinnotheridae). Zootaxa, 3528, 57–62.

Campos, E., & Vargas-Castillo, R. (2013). *Pinnotheres orcutti* Rathbun, 1918, a new Eastern Tropical Pacific species of *Tumidotheres* Campos, 1989 (Crustacea: Brachyura: Pinnotheridae). Zootaxa, 3666, 84–92.

Chopra, B. (1931). Further notes on Crustacea Decapoda in the Indian Museum. II. On some Decapoda Crustacea found in the cloaca of holothurians. Rec. Indian Mus., 33, 303–322.

Cuesta, J. A., Raso, J. E. G., Abelló, P., Marco-Herrero, E., Silva, L., & Drake, P. (2019). A new species of pea crab from south-western Europe (Crustacea, Decapoda, Brachyura): Species description, geographic distribution and population structure with an identification key to European Pinnotheridae. J. Mar. Biol. Assoc. UK, 99, 1141–1152.

Dai, A., & Yang, S. (1991). Crabs of the China Seas; China Ocean Press: Beijing, China; Springer: Berlin, Germany, pp. 421–437.

De Man, J. G. (1887). Report on the podophthalmous Crustacea of the Mergui Archipelago, collected for the Trustees of the Indian Museum, Calcutta, by Dr. John Anderson–Part II. J. Linn. Soc., 22, 65–128.

De Melo, G. A. S., & Boehs, G. (2004). Rediscovery of *Holothuriophilus tomentosus* (Ortmann) comb. nov. (Crustacea, Brachyura, Pinnotheridae) in the Brazilian coast. Rev. Bras. Zool., 21, 229–232.

Felder, D. L., & Palacios Theil, E. (2020a). A new pinnotherid crab of the genus *Pinnixulala* Palacios Theil, Cuesta & Felder, 2016 from uncertain infaunal hosts in the northern Gulf of Mexico, with a rediagnosis and updated synonymy for the polychaete symbiont *Pinnixulala retinens* (Rathbun, 1918) (Decapoda: Brachyura: Pinnotheridae). J. Crustacean Biol., 40, 887–898.

Felder, D. L., & Palacios Theil, E. (2020b). Three new symbiotic crabs of the genus *Glassella* Campos & Wicksten, 1997 from Atlantic and Gulf of Mexico coasts of Florida, USA (Decapoda: Brachyura: Pinnotheridae). J. Crustacean Biol., 40, 899–917.

Feldmann, R. M., Mackinnon, D. I., Endo, K., & Chirino-Galvez, L. (1996). *Pinnotheres laquei* Sakai (Decapoda: Pinnotheridae), a tiny crab commensal within the brachiopod *Laqueus rubellus* (Sowerby) (Terebratulida: Laqueidae). J. Paleontol., 70, 303–311.

Griffith, H. (1987). Taxonomy of the genus *Dissodactylus* (Crustacea: Brachyura: Pinnotheridae) with descriptions of three new species. Bull. Mar. Sci., 40, 397–422.

Hamel, J., Ng, P. K. L., & Mercier, A. (1999). Life cycle of the pea crab *Pinnotheres halingi* sp. nov., an obligate symbiont of the sea cucumber *Holothuria scabra* Jaeger. Ophelia, 50, 149–175.

Hernández-Ávila, I., & Campos, E. (2006). *Calyptraeotheres hernandezi* (Crustacea: Brachyura: Pinnotheridae), a new crab symbiont of the West Indian cup-and-saucer *Crucibulum auricula* (Gmelin) (Mollusca: Gastropoda: Calyptraeidae) off Cubagua Island, Venezuela. Proc. Biol. Soc. Wash., 119, 43–48.

Holthuis, L. B. (1975). *Limotheres*, a new genus of pinnotherid crab, commensal of the bivalve *Lima*, from the Caribbean Sea. Zool. Meded., 48, 291–295.

Hombron, J. B., & Jacquinot, H. (1842–1854). Atlas d’histoire naturelle Zoologie. Voyage au Pôle Sud et dans l’Océanie sur les corvettes l’Astrolabe et la Zélée pendant les années 1837–1838–1839–1840. Gide et J. Baudry, Paris.

Hsueh, P. W., & Huang, J. F. (1996). A new record of *Pinnotheres bidentatus* Sakai, 1939 (Decapoda: Brachyura: Pinnotheridae), from Taiwan. Crustacean Res., 25, 54–58.

Jiang, W., & Liu, R. (2011). New species and new records of pinnotherid crabs (Crustacea: Decapoda: Brachyura) from the Yellow Sea. Zool. Anz., 250, 488–496.

Kazmi, Q. B., & Manning, R. B. (2003). A new genus and species of pinnotherid crab from Karachi, northern Arabian Sea (Crustacea, Decapoda, Brachyura). J. Nat. Hist., 37, 1085–1089.

Komai, T., Nishi, E., & Taru, M. (2014). A new species of *Pinnixa* (Crustacea: Decapoda: Brachyura: Pinnotheridae) associated with a tube worm, *Chaetopterus cautus* (Annelida: Polychaeta), from Tokyo Bay, Japan. Zootaxa, 3793, 119–132.

Komatsu, H., & Ohtsuka, S. (2009). A new species of the genus *Abyssotheres* (Crustacea, Decapoda, Brachyura, Pinnotheridae) from the Ryukyu Islands, Southwestern Japan, with taxonomic notes on the genus. Bull. Natl. Mus. Nat. Sci. Ser. A Zool., 35, 73–81.

Manning, R. B. (1993a). *Epulotheres angelae*, new genus, new species, a pinnotherid crab from the Caribbean Sea (Decapoda: Pinnotheridae). J. Crustacean Biol., 13, 801–804.

Manning, R. B. (1993b). Three genera removed from the synonymy of *Pinnotheres* Bosc, 1802 (Brachyura: Pinnotheridae). Proc. Biol. Soc. Wash., 106, 523–531.

Manning, R. B. (1993c). West African pinnotherid crabs, subfamily Pinnotherinae (Crustacea, Decapoda, Brachyura). Bull. Mus. Natl. D’histoire Nat. Paris Ser., 15, 125–177.

Manning, R. B. (1996). *Viridotheres marionae*, a new genus and species of pinnotherid crab from West Africa (Crustacea: Decapoda: Brachyura). Zool. Meded., 70, 271–273

Manning, R. B. (1998). A new genus and species of pinnotherid crab (Crustacea, Decapoda, Brachyura) from Indonesia. Zoosystema, 20, 357–362.

Marin, I. N. (2014). Finding of the pea crab *Pinnaxodes mutuensis* Sakai, 1939 (Crustacea: Decapoda: Pinnotheridae) in an unusual host in Busse Lagoon, southern Sakhalin. Russ. J. Mar. Biol., 40, 486–489.

Miers, E. J. (1886). The Voyage of H.M.S. Challenger-Report of the Brachyura collected by H.M.S. Challenger during the years 1873–76. In Report on the Scientific Results of the Voyage of H.M.S.; Eyre & Spottiswoode: London, UK, pp. 1–362.

Ng, P. K. L. (2018). On the identities of *Pinnotheres villosissimus* Doflein, 1904, *P. dofleini* Lenz, in Lenz & Strunck, 1914, and *P. pilumnoides* Nobili, 1906 (Decapoda, Brachyura, Pinnotheridae) from the Western Indian Ocean. Crustaceana, 91, 611–633.

Ng, P. K. L., & Ahyong, S. T. (2022). The pea crab genus *Arcotheres* Manning, 1993 (Crustacea: Brachyura: Pinnotheridae) from Singapore and Peninsular Malaysia, with a reappraisal of diagnostic characters and descriptions of two new genera. Raffles Bull. Zool., 70, 134–248.

Ng, P. K. L., Ahyong, S. T., & Campos, E. (2019). Two new genera of pinnotherid crabs (Crustacea: Brachyura: Pinnotheroidea) from the Americas and the Western Pacific. Raffles Bull. Zool., 67, 337–351.

Ng, P. K. L., Clark, P. F., & Naderloo, R. (2022). Redescription of *Arcotheres tivelae* (Gordon, 1936), a pea crab endemic to the Persian Gulf and Gulf of Oman (Crustacea: Decapoda: Brachyura: Pinnotheridae). Zootaxa, 5141, 277–286.

Ng, P. K. L., & Ho, P. (2016a). A new genus for *Fabia obtusidentata* Dai, Feng, Song and Chen, 1980, a pea crab (Decapoda: Brachyura: Pinnotheridae) symbiotic with the moon scallop *Amusium* *pleuronectes* (Linnaeus, 1758) (Mollusca: Pectinidae). J. Crustacean Biol., 36, 740–751.

Ng, P. K. L., & Ho, P. (2016b). *Orthotheres baoyu*, a new species of pea crab (Crustacea: Brachyura: Pinnotheridae) associated with abalones from Tungsha Island, Taiwan; with notes on the genus. Raffles Bull. Zool., 64, 229–241.

Ng, P. K. L., & Kumar, A. B. (2015). A new species of *Afropinnotheres* Manning, 1993 (Crustacea, Brachyura, Pinnotheridae) from southwestern India, the first record of the genus from the Indian Ocean, with a review of the Pinnotheridae of India and adjacent seas. Zootaxa, 3947, 264–274.

Ng, P. K. L., & Manning, R. B. (2003). On two new genera of pea crabs parasitic in holothurians (Crustacea: Decapoda: Brachyura: Pinnotheridae) from the Indo-West Pacific, with notes on allied genera. Proc. Biol. Soc. Wash., 116, 901–919.

Ng, P. K. L., & Meyer, C. (2016). A new species of pea crab of the genus *Serenotheres* Ahyong & Ng, 2005 (Crustacea, Brachyura, Pinnotheridae) from the date mussel *Leiosolenus* Carpenter, 1857 (Mollusca, Bivalvia, Mytilidae, Lithophaginae) from the Solomon Islands. ZooKeys, 623, 31–41.

Ng, P. K. L., & Ngo, V. T. (2010). *Solenotheres prolixus*, a new genus and new species of pinnotherid crab (Crustacea: Decapoda: Brachyura) associated with the razor clam, *Solen corneus* Lamarck, 1818 (Solenidae) in Vietnam. Zootaxa, 2570, 61–68.

Ng, P. K. L., & Ngo, V. T. (2022). The Pinnotheridae (Decapoda, Brachyura) of Vietnam. Crustaceana, 95, 271–288.

Nobili, M. G. (1906). Diagnoses préliminaires de 34 espèces et variétés nouvelles, et de 2 genres nouveaux de décapodes de la Mer Rouge. Bull. Muséum D’histoire Nat. Paris Prem. Séries, 6, 393–411.

Page, R. D. M. (1983). Description of a new species of *Pinnotheres*, and redescription of *P. novaezelandiae* (Brachyura: Pinnotheridae). N. Z. J. Zool., 10, 151–162.

Palacios Theil, E., & Felder, D. L. (2019). Molecular phylogeography of *Tumidotheres maculatus* (Say, 1818) and *Zaops ostreus* (Say, 1817) (Crustacea: Decapoda: Pinnotheridae) in the western Atlantic, with description of a new species and synonymy of *Epulotheres* Manning, 1993. Mar. Biol. Res., 15, 548–567.

Palacios Theil, E., & Felder, D. L. (2020). Phylogeny of the genus *Austinixa* Heard & Manning, 1997, inferred from mitochondrial and nuclear molecular markers, with descriptions of three new species and redescription of *Austinixa felipensis* (Glassell, 1935) (Decapoda: Brachyura: Pinnotheridae). Zootaxa, 4778, 101–134.

Pregenzer, C. (Jr.) (1979). A redescription of *Pinnotheres hickmani* (Guiler) and comparison with *Pinnotheres novaezelandiae* Filhol and *Pinnotheres pisum* (L.) (Decapoda Brachyura). Crustaceana, Suppl. 5, 22–30.

Rahayu, D. L., & Ng, P. K. L. (2010). Two new species of *Indopinnixa* Manning & Morton, 1987 (Crustacea: Brachyura: Pinnotheridae) from Lombok, Indonesia. Zootaxa, 2478, 59–68.

Rathbun, M. J. (1918). The Grapsoid Crabs of America. Bull. U. S. Natl. Mus., 97, 1–461.

Rodrigues Costa, H. (1970). As espécies Brasileiras da família Pinnotheridae (Crustacea Reptantia) com descrição de uma nova espécie (*Fabia sebastianensis*). Trabalhos Oceanográficos da Universidade Federal do Pernambuco, Recife 9, 255–264.

Sakai, T. (1939). Studies on the Crabs of Japan IV; Yokendo: Tokyo, Japan, pp. 583–605.

Sakai, T. (1976). Crabs of Japan and adjacent seas. Plates. Kodansha Ltd: Tokyo, Japan, pl. 1–251.

Shen, C. J. (1932). The brachyuran Crustacea of North China. Zoologia Sinica, Peiping, (A), 9, i–x, 1–320.

Takeda, M., & Masahito, P. (2000). Systematic notes on the pinnotherid crabs of the genus *Pinnaxodes* (Crustacea: Decapoda: Brachyura). Bull. Natl. Sci. Mus. Tokyo, 26, 99–112.

Tesch, J. J. (1918). Siboga Expeditie: The Decapoda Brachyura of the Siboga Expedition I-Hymenosomidae. Retroplumidae, Ocypodidae, Grapsidae and Gecarcinidae; E.J. Brill: Leiden, The Netherlands, pp. 1–148.

Thoma, B. P., Heard, R. W., & Vargas, R. (2005). A new species of *Parapinnixa* (Crustacea: Brachyura: Pinnotheridae) from Isla del Coco, Costa Rica. Proc. Biol. Soc. Washington 118, 543–550.

Trivedi, J. N., Vachhrajani, K. D., & Ng, P. K. L. (2018). Redescription of *Arcotheres placunae* (Hornell & Southwell, 1909) (Crustacea: Decapoda: Brachyura: Pinnotheridae) from India and Pakistan. Zootaxa, 4433, 50–58.

Wells, H. W., & Wells, M. J. (1961). Observations on *Pinnaxodes floridensis*, a new species of pinnotherid crustacean commensal in holothurians. Bull. Mar. Sci. Gulf Caribb., 11, 267–279.

Zmarzly, D. L. (1992). Taxonomic review of pea crabs in the genus *Pinnixa* (Decapoda: Brachyura: Pinnotheridae) occurring on the California Shelf, with descriptions of two new species. J. Crustacean Biol., 12, 677–713.

**Appendix S3.** Morphospace plot showing the total variation of dorsal carapace shapes of both the in- and outgroups. Colours of points and convex hulls correspond to host association type (see Figure 3). Diamonds show the non-pinnotherine outgroups. Numbers correspond to the species in Appendix S2.


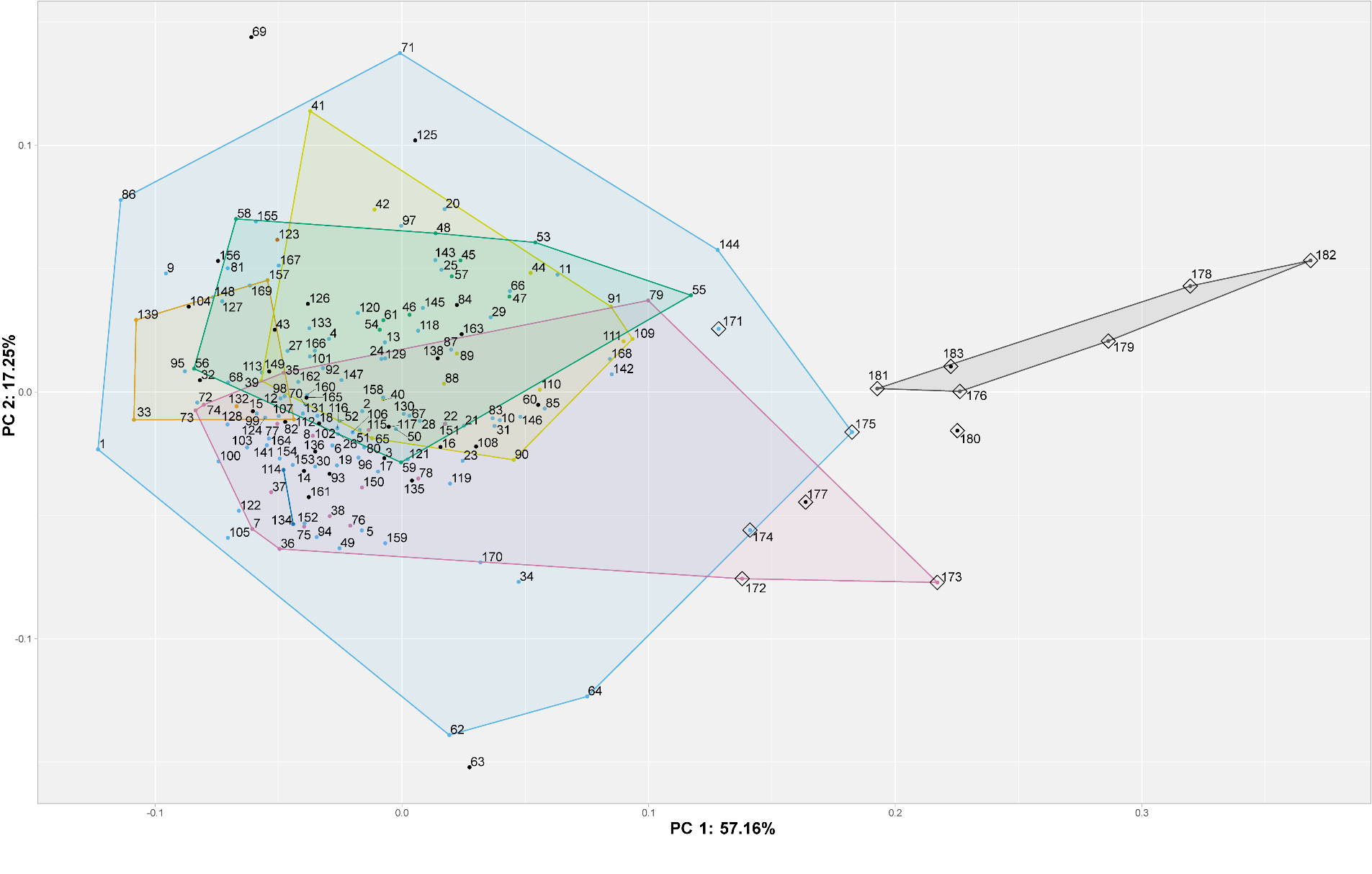

Supplement: Supplementary file 1 — Supinfo [file ECE3-13-e9744-s001.docx]
